# Combined transcriptomics and proteomics forecast analysis for potential genes regulating the Columbian plumage color in chickens

**DOI:** 10.1371/journal.pone.0210850

**Published:** 2019-11-06

**Authors:** Xinlei Wang, Donghua Li, Sufang Song, Yanhua Zhang, Yuanfang Li, Xiangnan Wang, Danli Liu, Chenxi Zhang, Yanfang Cao, Yawei Fu, Ruili Han, Wenting Li, Xiaojun Liu, Guirong Sun, Guoxi Li, Yadong Tian, Zhuanjian Li, Xiangtao Kang

**Affiliations:** 1 College of Animal Science and Veterinary Medicine, Henan Agricultural University, Zhengzhou, Henan, China; 2 College of Animal Science and Technology, Henan University of Animal Husbandry and Economy, Zhengzhou, Henan, China; Nazarbayev University, KAZAKHSTAN

## Abstract

**Background:**

Coloration is one of the most recognizable characteristics in chickens, and clarifying the coloration mechanisms will help us understand feather color formation. “Yufen I” is a commercial egg-laying chicken breed in China that was developed by a three-line cross using lines H, N and D. Columbian plumage is a typical feather character of the “Yufen I” H line. To elucidate the molecular mechanism underlying the pigmentation of Columbian plumage, this study utilizes high-throughput sequencing technology to compare the transcriptome and proteome differences in the follicular tissue of different feathers, including the dorsal neck with black and white striped feather follicles (Group A) and the ventral neck with white feather follicles (Group B) in the “Yufen I” H line.

**Results:**

In this study, we identified a total of 21,306 genes and 5,203 proteins in chicken feather follicles. Among these, 209 genes and 382 proteins were differentially expressed in two locations, Group A and Group B, respectively. A total of 8 differentially expressed genes (DEGs) and 9 differentially expressed proteins (DEPs) were found to be involved in the melanogenesis pathway. Additionally, a specifically expressed *MED23* gene and a differentially expressed GNAQ protein were involved in melanin synthesis. Kyoto Encyclopedia of Genes and Genomes (KEGG) analysis mapped 190 DEGs and 322 DEPs to 175 and 242 pathways, respectively, and there were 166 pathways correlated with both DEGs and DEPs. 49 DEPs/DEGs overlapped and were enriched for 12 pathways. Transcriptomic and proteomic analyses revealed that the following pathways were activated: melanogenesis, cardiomyocyte adrenergic, calcium and cGMP-PKG. The expression of DEGs was validated by real-time quantitative polymerase chain reaction (qRT-PCR) that produced results similar to those from RNA-seq. In addition, we found that the expression of the *MED23*, *FZD10*, *WNT7B* and *WNT11* genes peaked at approximately 8 weeks in the “Yufen I” H line, which is consistent with the molting cycle. As both groups showed significant differences in terms of the expression of the studied genes, this work opens up avenues for research in the future to assess their exact function in determining plumage color.

**Conclusion:**

Common DEGs and DEPs were enriched in the melanogenesis pathway. *MED23* and GNAQ were also reported to play a crucial role in melanin synthesis. In addition, this study is the first to reveal gene and protein variations in in the “Yufen I” H line during Columbian feather color development and to discover principal genes and proteins that will aid in functional genomics studies in the future. The results of the present study provide a significant conceptual basis for the future breeding schemes with the “Yufen I” H line and provide a basis for research on the mechanisms of feather pigmentation.

## Introduction

“Yufen I” is an egg-laying chicken breed in China, which was developed by a three-line cross using line H as the first male parent, line N as the maternal grandparent and line D as the final male parent. As authorized by the National Commission on Livestock and Poultry Genetic Resources in 2015, this breed has been bred true for at least six generations. However, a closed breeding method was used to develop the line H by crossing the barred plumaged-original Gushi chicken with an egg-laying grandparent line C, the brown-shelled Babcock B-380. Line H is a fast plumage line and is characterized by early maturity, high egg production and the Columbian plumage pattern. Columbian plumage is a character of feather color in the “Yufen I” H line in which the dorsal neck, tail and apex of the wing feathers have black and white stripes and other feathers are white. This pattern was also regarded as the main pigmentation character of the “Yufen I” H line.

Complex feather coloration is likely coordinated through multiple genes that regulate diverse mechanisms, with more than 200 genes involved in pigmentation that have been studied in mammals [1]. Many studies have identified key genes that determine the types of pigments (melanin) that are expressed by melanocytes [2]. Birds are among the most colorful vertebrates. Melanins, porphyrins, polyenes, carotenoids and structural colors have been discovered in feathers [3]. Feather color in chickens is a result of the melanin produced by the melanocytes of the feather follicles. Feathers and feather follicles are ideal tissues to explore the genetic mechanisms and complexity of color patterns in birds. As a derivative of chicken skin, the feather follicles give rise to the feathers and are capable of self-renewal, and their proliferation and differentiation result in feather formation [4–6]. In adult feather follicles, plumage pigmentation is mainly dependent on the interaction between feather follicle melanocytes and dermal papilla fibroblasts. Pigmentation activity occurs only during the growth period of feather follicles, and the transfer of melanin and pigment to keratinocytes depends on the activity of melanin precursors. The melanin or pigment is transferred to the skin and feather follicles through the regulation of signal transduction pathways [7,8]. Studies have revealed that many growth factors and receptors coordinate genes and that the environment and signaling pathways play an extremely important role during feather growth. To date, some of the confirmed pathways involved in pigmentation include cAMP pathway [9], SCF-KIT pathway [10], PI3K-Akt pathway [11], BMP signaling pathway [12], Notch pathway [13], ERK pathway [14], CREB/MITF/tyrosinase pathway [15], MCIR/(Gs-AC)/PKA pathway [16–18], Wnt/β-catenin pathway [19,20] and MAPK pathway [21,22].

Uniformity in the appearance of birds is essential in the poultry industry [23]. Although plumage color is easily observed, the genetics behind feather pigmentation are governed by both qualitative and quantitative features [24]. Research reveals that the ratio of pheomelanin (yellow-red pigment) and eumelanin (black-brown pigment) pigmentation regulates feather color in chickens [25,26]. Eumelanin pigmentation requires that the melanoblasts migrate to the epidermis from the neural crest to ultimately reach the developing feather follicles. Another requirement is the proportion of the pigment subject to control by genes [27,28]. Melanosomes are responsible for synthesizing these pigments. These organelles are granule-like and develop within melanoblasts, which go through several steps of differentiation to form melanocytes. The preliminary steps for melanin production include the appearance of the neural crest, determination of melanoblasts, migration, proliferation and differentiation [29–31]. Besides this, melanogenesis regulation after the melanocytes migrate into feather follicles is another important step. The variation in plumage may be caused by any mutations in relevant genes and changes in molecules (receptors of transcription factors on cells, structural proteins, enzymes and growth factors) involved in the aforementioned process [32,17,33]. Until now, several studies have focused on genes such as *MC1R*, *ASIP*, *TYR*, *SLC24A5*, *KITLG*, *MITFCDKN2A/B*, *PMEL17* and *DCT*, which play a role in melanin proportion synthesis [34,25,35–41].

Many genes and pathways have been shown to be associated with pigmentation. However, research on the genetics of Columbian plumage in chickens is lacking. Feathers have different forms in terms of color and morphology, not only among different bird species but also among different body regions of an individual bird [42,43]. Some studies used transcriptomics to analyze the skin and hair follicles of poultry and found differentially expressed genes (DEGs) related to feather color and morphology, which provided a theoretical basis for studying the formation, development and regeneration of feathers [44,45]. In this experiment, we collected the feather follicles in two parts: the dorsal feather follicles of the neck with black and white-striped feathers (Group A) and the ventral feather follicles of the neck with white feathers (Group B). This study aimed to discover the differentially expressed genes (DEGs), differentially expressed proteins (DEPs) and Kyoto Encyclopaedia of Genes and Genomes (KEGG) pathways by transcriptomic (RNA-seq) and proteomic (iTRAQ) analyses and to forecast potential candidate genes of Columbian plumage in order to determine which genes and pathways affect feather coloration.

## Materials and methods

### Disclosure of ethics

Experimental animals were maintained per the rules in National standards for the environment and facilities of experimental animals of China (GB14925-2010). All the chickens were healthy, with a coop size of 0.12m^2^ and 0.4m high for each. Adequate and clean drinking water and feed were provided. All protocols for animal experiments received approval from the Institutional Animal Care and Use Committee (IACUC) of Henan Agricultural University and from Henan Agricultural University’s Animal Care Committee, College of Animal Science and Veterinary Medicine, China (Permit Number: 17–0322).

### Animals for experiments and tissue sources

**Fig 1:**
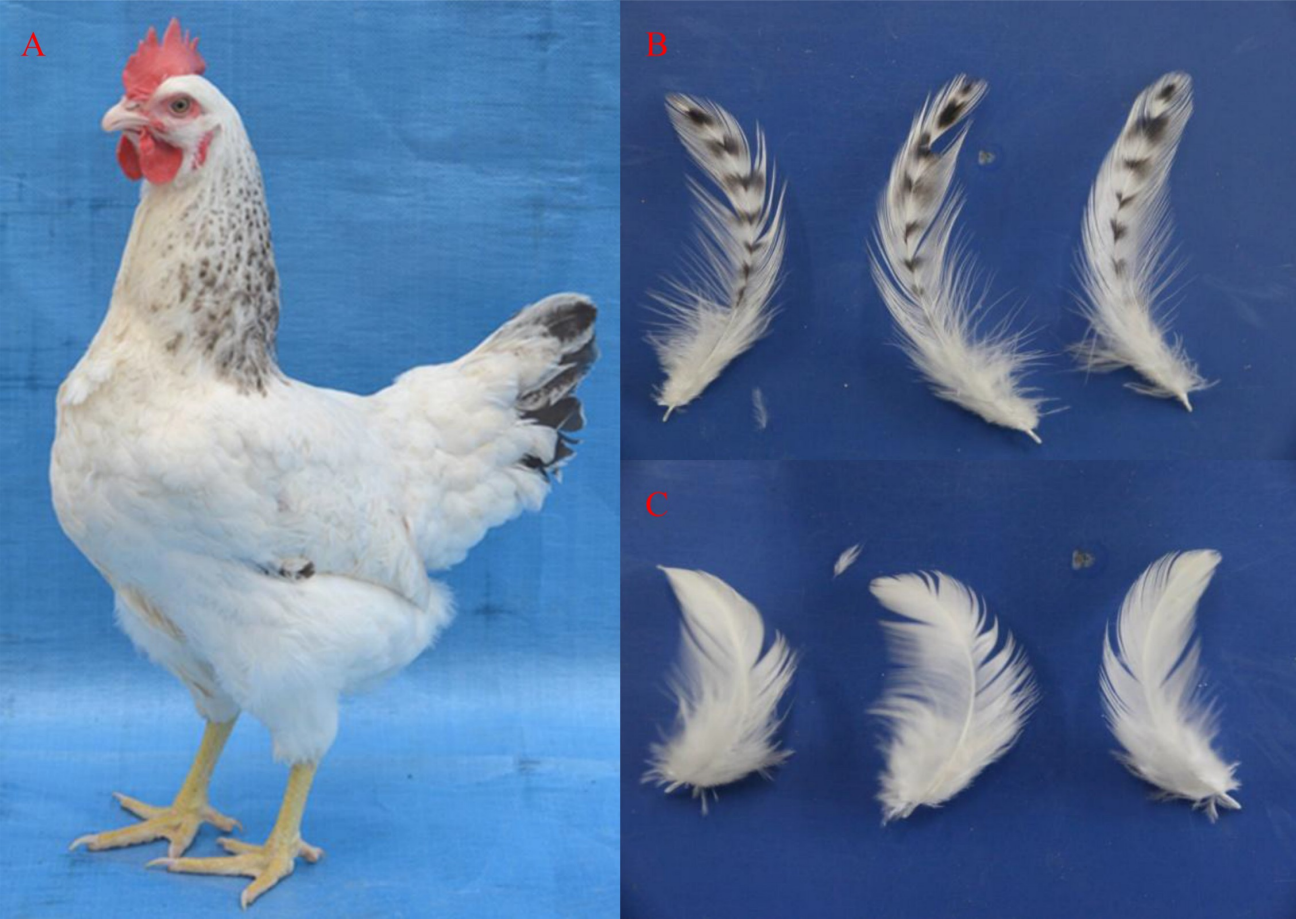
“Yufen I” H line chicken and the feather bulbs used in this study. **(**A). “Yufen I” H line chicken, (B). black and white striped-feathers in the dorsal neck, (C). white feathers in the ventral neck.

The Animal Center of Henan Agricultural University provided us with chickens of the “Yufen I” H line breed for this study. Three 22-week-old female chickens were anaesthetized with “Su-Mian-Xin” (a type of anesthetic, Shengda Animal Pharmaceutical Co., Ltd, Dunhua, China), which is diluted with saline at a volume ratio of 1:2. The chickens were anesthetized by i.v. injection in the wing vein with a concentration of 0.2mL/kg for approximately 20 minutes, and then the neck feathers were plucked. New feathers emerged in the skin after two weeks. Next, the three chickens involved in this study were placed in an airtight box and humanely sacrificed by inhaling carbon dioxide in order to reduce their suffering. Six tissue samples including feather follicles and the circumjacent skin closely around the rachis of the feathers were collected from the dorsal and ventral areas, the locations at which black and white-striped feathers and white feathers occur, respectively (Fig 1). Three replicates were collected for each group (A1 to A3 for Group A and B1 to B3 for Group B). Approximately 20~30 feather follicles were placed into 2-mL tubes, and then the tubes were sealed, dipped in liquid nitrogen to freeze quickly and stored at -80°C storage isolation and sequencing of RNA and qRT-PCR analysis [46].

### Extraction of total RNA and RNA-seq

Six frozen samples corresponding to the two feather follicle areas (Group A and Group B) were selected for isolation of total RNA. The feather follicles of the “Yufen I” H line were used to extract total RNA using TRIzol reagent (Invitrogen, USA) and used in library construction. The assessment of contamination and degradation of RNA were performed with standard denaturing agarose gel electrophoresis. The RNA integrity, concentration and purity were evaluated on an Agilent RNA6000 Nano Chip in Reagent Port 1 of the Bioanalyzer Agilent 2100 (Agilent Technologies, CA, USA). Sequencing of the libraries was performed on the Illumina HiSeq 4000 platform by BGI Co., Ltd. Data were deposeted in the NCBI Sequence Read Archive under Accession SRR7973871. To process the raw data, the FASTQ format was first processed through SOAPnuke (v1.5.2), and then reads carrying adapters, poly-N sequences and those of low quality were deleted from the raw data to obtain clean data. Additionally, we calculated the Q20 and Q30 at error rates of 1% and 0.1%, respectively, for the clean data. To ascertain whether resequencing was required, quality control (QC) for alignment was carried out. These high quality data were used for the analyses performed downstream.

### Transcriptomic data processing

After read filtering, we used HISAT (v0.1.6-beta) to perform genome mapping. To map RNA-seq reads, a spliced alignment program, HISAT, is fast and sensitive with an accuracy that is equal to or better than that of other methods. The spliced mapping algorithm of HISAT has been applied for genome mapping of preprocessed reads [47,48]. Reads that passed the QC test were aligned to a reference genome assembly of chicken (*Gallus gallus 5*.*0*, *https*:*//www*.*ncbi*.*nlm*.*nih*.*gov/assembly/GCF_000002315*.*4/*) from NCBI. Measurement of the abundance of expression for each assembled transcript was done using the Fragments per Kilobase of exon model per Million mapped reads (FPKM) values.

$$FPKM=\frac{total\;exon\;fragments}{mapped\;reads\;(millions)\times exon\;length\;(kb)}$$

For the two groups, to analyze differential expression, the levels of gene and transcript expression were determined using RSEM software (V1.2.12). For each pair of samples, the DEGs were screened using a model derived from PossionDis software [49]. In this paper, “false discovery rate (FDR*)* ≤ 0.001 and the fold change ≥ 2 (absolute value of log2Ratio ≥ 1)” were set as the significant threshold values to ascertain the differences between the gene expression. The phyper function of R software was used to identify enriched KEGG (http://www.kegg.jp/) pathways (p ≤ 0.05) and Gene Ontology (GO: http://www.geneontology.org), respectively. Additionally, to identify the major metabolic and signal transduction pathways, KEGG enrichment analysis was performed on the DEGs. Likewise, GO enrichment analysis was performed to ascertain the main molecular functions, cellular components and biological processes associated with DEGs.

### RNA validation through qRT-PCR

To validate the RNA-seq data, qRT-PCR was used, and seven DEGs were selected for the analysis. The same samples used for RNA sequencing were used for reverse transcription and synthesis of cDNA with the PrimeScript RT Reagent Kit with gDNA Eraser following the instructions of the manufacturer (TaKaRa, Dalian, China). To design the primers for qRT-PCR, the NCBI Primers-BLAST online program was used. Information regarding the primers of these genes can be found in S1 Table. Each 10 μL qRT-PCR reaction contained 1.0 μL of cDNA, 0.5 μL of each primer at 10 μM, 5.0 μL of 2×SYBR® Premix Ex Taq™ II (TaKaRa, Dalian, China), and 3 μL of deionized water. The reaction was carried out on a LightCycler® 96 Real-Time PCR system (Roche Applied Science, Indianapolis, USA).The internal control was the GAPDH gene, and the 2^-ΔΔCT^ method was used to assess expression. The procedure for qRT-PCR amplification is shown: 95°C for 3 min; 35 cycles at 95°C for 30 s, 60°C for 30 s, and 72°C for 20 s; and a final extension for 10 min at 72°C. The data were statistically analyzed using SPSS V 21.0 (SPSS Inc., Chicago, IL, USA). One-way and repeated- analyses of variance followed by Dunnett’s test were carried out. The data are shown in the form of the mean ± SE with significance set at p≤ 0.05.

### Protein extraction and iTRAQ reagent labeling

In the context of several experiments, an approach called isobaric tags for relative and absolute quantification (iTRAQ) has been successfully used. Here, the samples were used for RNA-seq as well as iTRAQ analysis. For protein extraction, the lysis of 2 g of each of the samples was carried out in lysis buffer 3 (TEAB with 1 mM PMSF, 8 M Urea, 10 mM DTT and 2 mM EDTA, pH 8.5) with 2 magnetic beads of 5 mm diameter. The samples were kept in a tissue lyser for 2 min at 50 Hz so that the proteins were released and then subjected to centrifugation at 25,000 x g for 20 min at 4 C, The the supernatant was transferred into a fresh tube, and 10 mM DTT (dithiothreitol) was added for reduction at 56°C for 60 mins, followed by alkylation using 55 mM IAM (iodoacetamide) at room temperature, in the dark for 45 min, and then centrifuged under the same conditions described above.

The concentration and quality of proteins were assessed with a Bradford assay of the supernatant and confirmed with 12% SDS-PAGE.Then, 100 mM TEAB was used to dilute 100μg of protein solution with 8M urea that was subjected to digestion using Trypsin Gold (40:1 protein: enzyme, Promega, Madison, WI, USA) at 37°C overnight. Subsequently, an iTRAQ Reagent 8-plex Kit was used to label samples, followed by combining the differently labeled peptides, desalting using a Strata X C18 column (Phenomenex), and finally vacuum drying in accordance with the instructions in the manufacturer's protocol. After this step, fractionation of these pooled mixtures was performed on a Shimadzu LC-20AB high pressure liquid chromatography (HPLC) pump system with a high pH RP column, followed by nanoelectrospray ionization and subsequent tandem mass spectrometry (MS/MS) on a Triple TOF 5600 system (SCIEX, Framingham, MA, USA). Triplicate experiments were carried out.

### Proteomics database processing

The ProteoWizard tool was utilized to convert raw MS/MS data into “.mgf” format followed by searching these exported files with Mascot (v 2.3.02) in this project against a database (*Gallus gallus 5*.*0*, https://www.ncbi.nlm.nih.gov/assembly/GCF_000002315.4/). Identification required the presence of a minimum of one unique peptide. Parameters set included: MS/MS ion search; Trypsin enzyme; 0.1 Da was the mass tolerance of fragment; Monoisotopic mass values; Variable modifications oxidation (M) and iTRAQ8plex (Y); 0.05 Da was the tolerance for Peptide mass; Fixed modifications Carbamidomethyl (C), iTRAQ8plex (N-term), iTRAQ8plex (K); Database I-ZAwBa007 (50596 sequences); Database_info transcriptome. Identification was performed using peptides that reached a confidence level of 95%.

The iTRAQ peptides were analyzed in a quantitative format with IQuant automated software [50]. This software incorporates a method based on machine learning called Mascot Percolator [51], which can rescore results from databases to yield a significant scale of standards. A 1% FDR was used to pre-filter PSMs that were used to measure the confidence level. A set of confident proteins was assembled using the parsimony principle also termed as the “simple principle”. Using the picked protein FDR strategy as a basis [52], followed by protein inference with an FDR at a protein level lesser than or equal to 0.01, an FDR of 1% was measured in order to limit the number of false positives. The quantification of proteins involved identification of proteins, correction of tag impurities, normalization of data, imputation of missing values, calculation of protein ratio, and statistical approaches followed by presentation of the results.

The proteins that had a p-value lower than 0.05 and a fold change of 1.2 were classified as DEPs. Analysis of metabolic pathways was in accordance with KEGG, while analysis of other databases, COG and GO, was in lieu of earlier research [53]. The hypergeometric test was applied to analyze DEP enrichment analysis in KEGG and GO. The equation was as follows:

p=1−∑i=0m−1(Mi)(N−Mn−i)(Nn)

Where *N* represents the quantity of identified proteins that were linked to data from GO and KEGG analyses, *n* represents the measure of DEPs within *N*, *M* represents the quantity of proteins linked to a pathway or term of GO or KEGG, and *m* represents the quantity of DEPs linked to a pathway or term of GO or KEGG. An enrichment of differential proteins was considered when the p-value ≤ 0.05. The DEG analysis was the same as the DEP analysis.

### Transcriptomics and proteomics: Association analysis

Genes are regulated at multiple levels during the expression process. At present, most studies have reported that the expression consistency between mRNAs and their corresponding proteins is not very high, so a combined analysis of the proteome and transcriptome is helpful for discovering the regulation of gene expression [54]. Cluster 3.0 software was utilized to analyze clusters for DEP expression with that of transcripts in order to recognize transcripts and DEPs with similarity across various tissues with a graphical output from Java Treeview software. All expression data related to proteomics and transcriptomics were analyzed, and the Spearman correlation coefficient was calculated using R software [55]. Associations between the expression of mRNA and proteins from these respective “omics” were quantified followed by analysis for GO as well as KEGG enrichmentin order to study the potential role of DEGs/DEPs in metabolism or signal transduction. The combined transcriptomic and proteomic analysis parameters are given in S2 Table.

## Results

### Analysis of data from RNA sequencing

The Illumina HiSeq 4000 platform was employed to sequence 6 preparations of cDNA library. The RNA-seq data showed high consistency among the libraries (Table 1). A total of 44.06 MB of clean reads and 45.05 MB of clean reads were obtained from the 6 cDNA libraries. The Q30 for each sample was more than 94%, and approximately 71% of the clean reads were mapped to the chicken genome (Gallus *gallus 5*.*0*).The data are indicative of data of high quality sequencing which can ensure the reliability of results for further investigation.

**Table 1:** Gene expression and clean reads analyses in “Yufen I” H line feather follicles.

Sample name	Raw reads/Mb	Clean reads/Mb	Clean reads Q30/%	Mapping rate/%	Uniquely mapped rate/%	Total gene number	Total transcript number
A1	66.38	44.06	94.88	74.23	67.56	19054	36729
A2	69.62	45.05	94.74	71.66	62.18	18640	33464
A3	72.86	44.23	95.00	70.56	63.28	18669	33808
B1	66.38	44.90	94.74	71.65	65.75	18567	33874
B2	69.62	44.18	94.84	67.83	62.22	18565	34097
B3	66.38	44.62	94.80	71.97	64.73	18793	33858

### Identification of DEGs

To identify genes involved in feather coloration, DEGs were detected in the “Yufen I” H line using PossionDis software [56]. Of a total of 21,306 identified genes, 597 novel genes and 12,687 novel isoforms were identified in chicken feather follicle libraries in this work. A total of 209 (83 upregulated and 126 downregulated) DEGs were detected at the two locations (the dorsal and ventral areas, where black and white-striped feathers and white feathers occur). All of the DEGs are illustrated in S3 Table. Earlier research showed several genes involved in pigmentation (e.g., *ASIP*, *KITLG*) [57,58].

**Fig 2:**
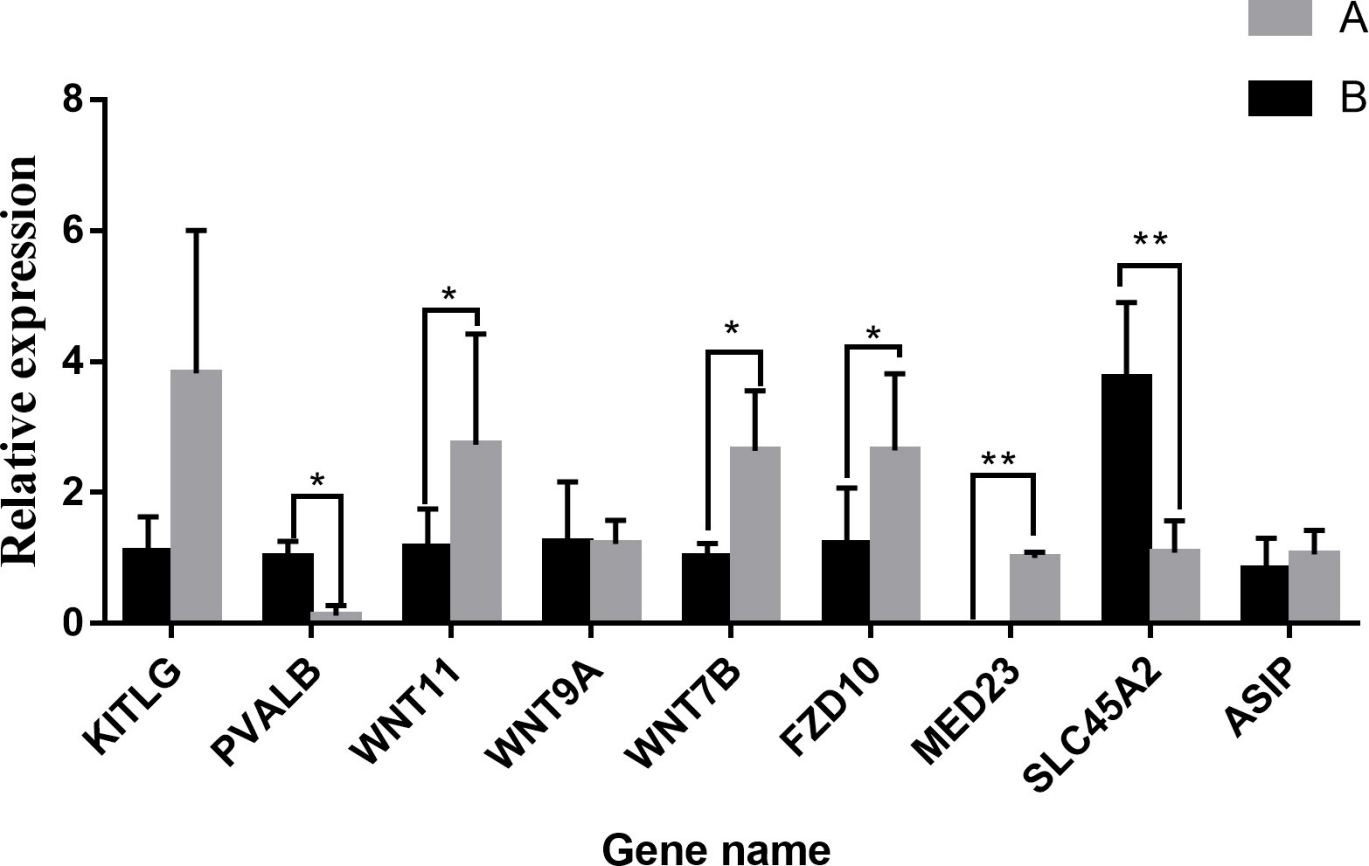
Verification of DEGs via qRT-PCR. The 2^-ΔΔCT^ method was used for data analysis, and the housekeeping gene was GAPDH. Data shown on the vertical-axis represents the relative expression. Significant differences between two groups were determined by applying the unpaired Student’s t-test. * 0.01 < p < 0.05, ** p < 0.01. All dataare presented as means ± standard error (SE).

In order to corroborate whether the RNA-seq gene expression data were accurate and reproducible, 7 genes were subjected to selection for qRT-PCR in the two groups. The expression of *KITLG*, *FZD10*, *WNT7B*, *WNT9A*, *WNT11*, *PVALB* and *MED23* was validated by qRT-PCR (Fig 2). As the results from both analyses were consistent with each other, the reliability of the sequencing was indicated.

**Fig 3:**
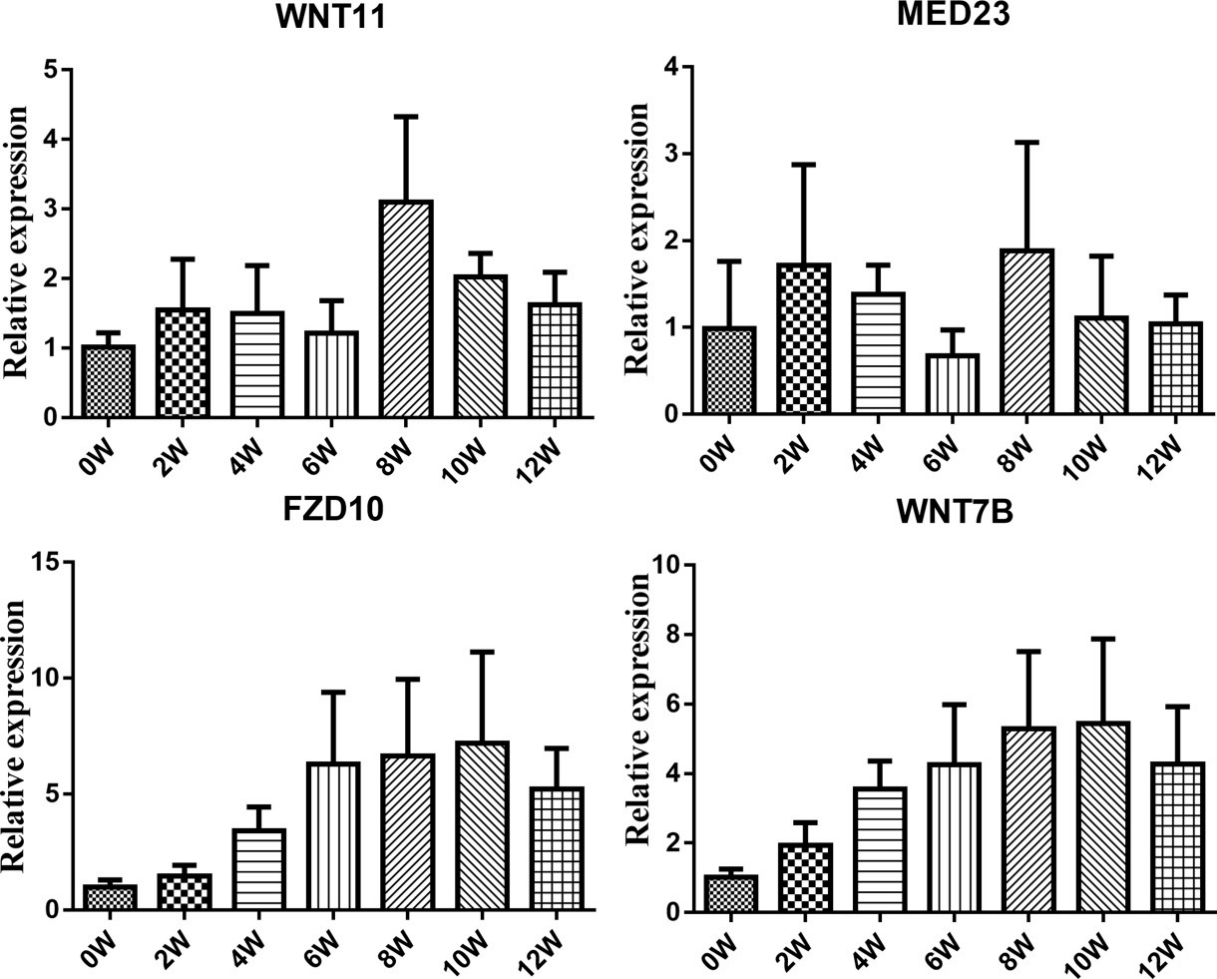
qRT-PCR validation of DEGs involved in the feather cycle. Changes in DEGs in feather follicles from the dorsal neck of the chicken at 0, 2, 4, 6, 8, 10 and 12 weeks.

The downy feathers of chickens are reportedly replaced by the second generation at the age of 6 weeks. At the age of 8 weeks, the second generation of feathers begins to molt and a large number of third-generation feathers begin to appear. At this time, the feather color tends to be stable. We collected feather follicles at 0, 2, 4, 6, 8, 10 and 12 weeks from the dorsal neck of the “Yufen I” H line chicken. Using qRT-PCR, we found that *MED23*, *FZD10*, *WNT7B* and *WNT11* gene expression peaked at approximately 8 weeks, which is consistent with the molting cycle (Fig 3). Furthermore, *FZD10*, *WNT7B and WNT11* are located in the melanogenesis pathway, and *MED23* is specifically expressed in the dorsal feather follicles of the neck with black and white-striped feathers.

### Metabolic pathways and GO analysis of DEGs

KEGG pathway and GO analyses were performed in order to interpret the exact roles played by the DEGs that regulate feather pigmentation. Using Blast2GO, we classified the DEGs with GO terms and KEGG pathways according to their functions. With the KEGG and GO annotation results, official classification of DEGs was performed followed by functional enrichment study with these databases using phyper, an R software function.

**Fig 4:**
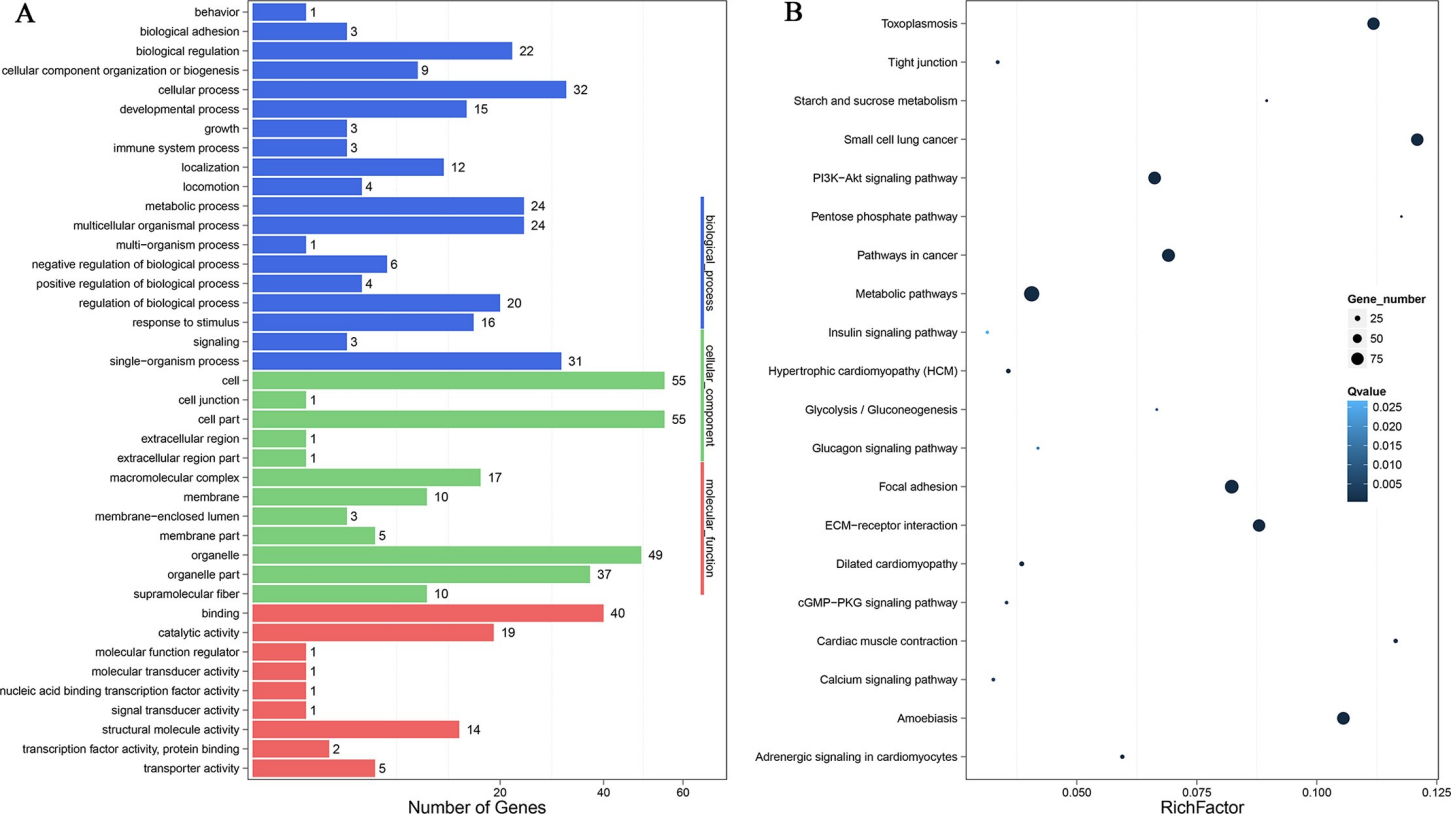
KEGG and GO analyses of DEGs. (A) GO functional annotation histogram of the DEGs. The vertical axis represents the three GO categories, the horizontal axis represents the gene number, and the number of genes is considered the difference in the proportion of the total. The GO annotations are classified in three basic categories, including cellular component, biological processes, and molecular function. (B) The degree of enrichment of the first 20 entries in the pathway. The pathway names are represented on the vertical axis, and the horizontal axis represents the pathways corresponding to the rich factor. The ratio of the number of DEGs and all annotated genes in the pathway is defined as the rich factor.

Classification of 209 DEGs with GO terms was performed with the Blast2GO platform to identify functional roles with an abundance of the following terms: “biological processes,” “molecular functions” and “cellular components” (FDR ≤ 0.01). Many genes were associated with cell part, cell, organelle, and cellular process as well as binding. The categories with abundance were the processes associated with cell, single-organism, metabolic process and multicellular organisms, as well as regulation of biological process in the biological processes category. Cells, cell part and organelles showed maximum abundances in the category called cellular component. The majority of the unigenes could be classified into binding and catalytic activity functions in terms of function (Fig 4A).

As a result, 190 DEGs were mapped to 175 pathways in KEGG (FDR ≤ 0.01); therein, 188 DEGs were enriched in environmental information processing, of which 81 DEGs were enriched at the level of signaling molecules and interaction and 107 DEGs showed enrichment at the level of signal transduction. The most enriched pathways of the DEGs were focal adhesion, metabolic pathways, PI3K-Akt, cGMP-PKG, calcium and adrenergic signaling pathway in cardiomyocytes showed significant enrichment in two groups (Fig 4B).

### iTRAQ data analysis

iTRAQ analysis or proteomics was carried out to supplement the transcriptomics data from the same samples. The range of masses of identified proteins was 10 to 100 kDa, while the average coverage was challenged for groups with more than 100 kDa. These data are indicative of reliable proteomic analyses [59]. This technique aided the identification of 293,356 spectra in the samples which were subjected to data filtering to obtain 51,631 unique spectra that could be matched with 23,244 unique peptides, for the total identification of 5,203 proteins (Table 2).

**Table 2:** Information on identified proteins in chicken feather follicles.

Sample name	Total spectra	spectra	Unique Spectra	Peptide	Unique Peptide	Protein
Yufen I	293,356	60,886	51,631	25,545	23,244	5,203

### Functional classification and annotation of DEPs

**Fig 5:**
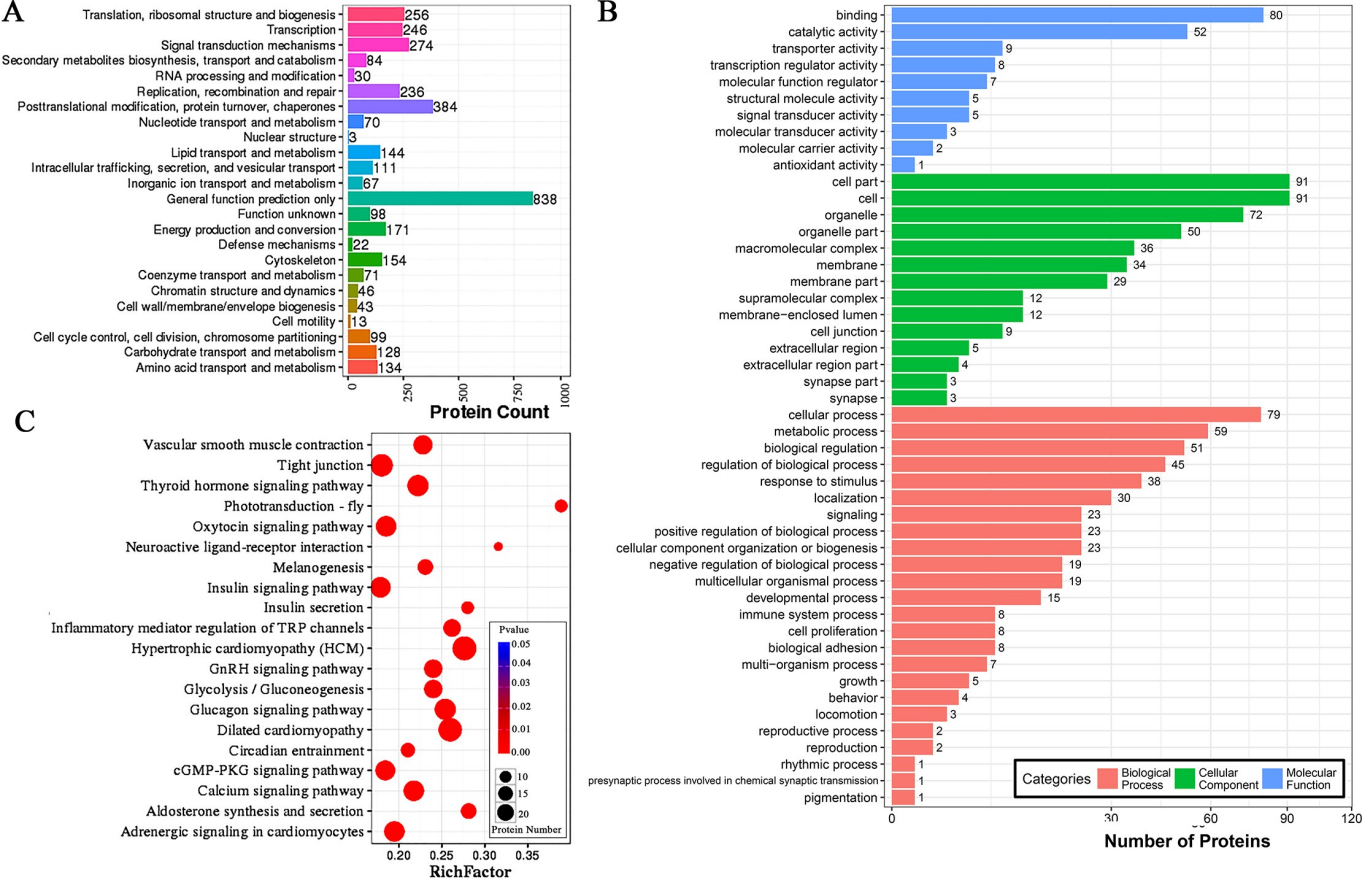
DEP analysis in terms of GOG, GO and KEGG. (A) The horizontal axis represents the COG term, and the vertical axis represents the corresponding protein count illustrating the protein number of different function. (B) GO functional annotation histogram of the DEPs. The three GO categories are presented on the vertical axis under the GO term, the horizontal axis represents the gene number, and the number of genes is accounted for by differences in the proportion of the total. The GO annotations are classified in three basic categories including cellular component, biological processes, and molecular function. (C) The name of the pathway is mentioned on the vertical axis, and the pathway matching the rich factor are mentioned on the horizontal axis. The rich factor is the ratio of the number of DEPs in the pathway to the number of all annotated proteins in the pathway. After testing multiple hypotheses, Q values were completed with corrected P value in the range of 0–0.05. The enrichment was considered significant if the P-value was closer to zero.

The DEPs were identified with a p-value < 0.05 and fold change ≥ 1.2 between the dorsal and ventral feather follicles of the neck. In brief, 382 DEPs (160 upregulated and 222 downregulated) were detected in the feather follicles (S4 Table), followed by classification into functions of COG categories. Clearly, changes were observed in the levels of proteins involved in functions such as general function prediction only; posttranslational modification, protein turnover; signal transduction mechanisms; translation, ribosomal structure and biogenesis; transcription and cytoskeleton (Fig 5A). DEPs were subjected to enrichment and clustering using functional analyses of GO and KEGG. In the GO function analysis, cell part, intracellular part, and cytoplasm of cellular component; binding, catalytic activity and ion binding of molecular function; and biological regulation, response to stimulus and metabolic process of biological processes, were found to be different between the samples (Fig 5B). Regarding KEGG pathway functions associated with the feather follicles, significant differences were found in the following signaling pathways: calcium, PI3K-Akt, mTOR, cAMP, MAPK, Wnt, cGMP-PKG, Jak-STAT and adrenergic signaling in cardiomyocytes, melanogenesis, tyrosine metabolism, and melanoma. We displayed the top 20 enriched pathways in Fig 5C. At present, under feather regeneration treatment, segment polarity protein disheveled homolog DVL-3 isoform X6 protein (DVL3), 1-phosphatidylinositol 4,5-bisphosphate phosphodiesterase beta-1-like protein (LOC107052863), 1-phosphatidylinositol 4,5-bisphosphate phosphodiesterase beta-1 protein (PLCB1), GTPase KRas isoform X2 protein (KRAS), matrix-remodeling-associated protein 8 precursor protein (MXRA8), guanine nucleotide-binding protein G(q) subunit alpha protein (GNAQ), parvalbumin muscle isoform X1 protein (PVALB), calcium/calmodulin-dependent protein kinase type II subunit beta protein (CAMK2B) and calcium/calmodulin-dependent protein kinase type II subunit alpha isoform X3 protein (CAMK2A) were differentially expressed in the dorsal follicles of the neck compared with the ventral follicles of the neck and were mainly enriched for the melanogenesis pathway. The results indicated that upon plucking stimulation, the DEPs related to feather pigmentation were mainly concentrated in the melanogenesis pathway.

### Integrating transcriptomic and proteomic results

A majority of earlier reports are suggestive of a weak correlation between the expression of mRNA with protein attributed to posttranscriptional regulation or posttranslational modification or experimental errors [60–62]. Nevertheless, the flow of information from RNA to proteins is the crux of the central dogma [63,64]. To allow for genes that are expressed differentially in terms of the transcriptome and proteome as well as singling out vital genes, we performed an integration of DEGs and DEPs. For multi-omics data, GO terms and KEGG pathways were enriched at the levels of transcriptome and proteome, respectively. Then, the data from the two groups were integrated and analyzed, which was conducive to the study of gene expression regulation at the level of gene set coexpression [65–68].

**Fig 6:**
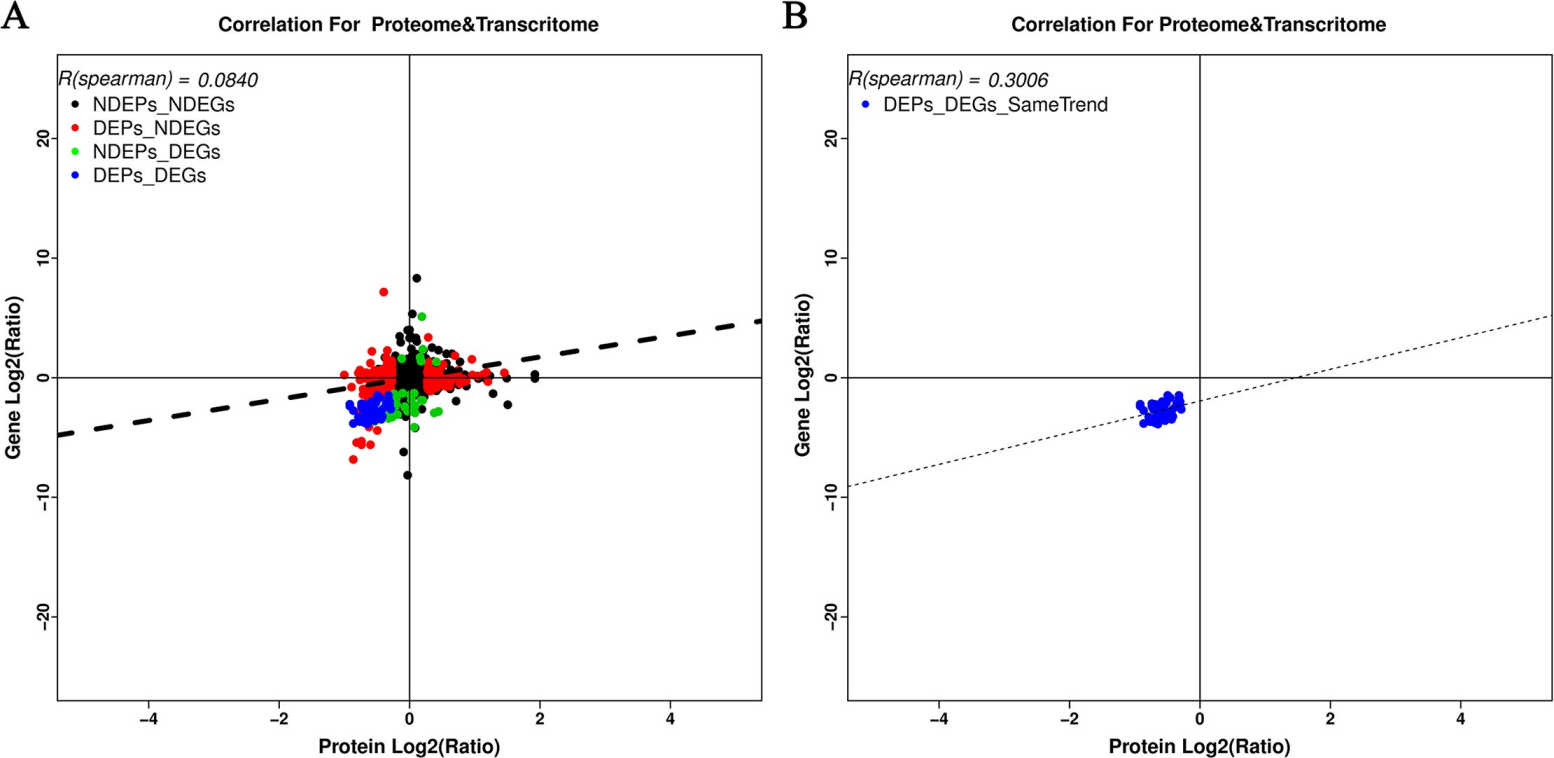
Correlations between the expression of proteins and genes. The vertical-axis represents the protein expression level, and the horizontal-axis represents the genes expression level. (A) Scatter plots of the correlation between data sets of genes evaluated in both the proteomic and gene transcript analyses. (B) Scatter plots and coefficients of DEPs and DEGs correlation.

To compare the proteomic and transcriptomic analyses, we compared the 382 DEPs with the 209 DEGs. According to the results, only 49 genes meeting the criteria overlapped (S5 Table). The changes at the transcript and protein levels showed a weak correlation for the proteins that were quantified. Biological pathways were elucidated with a change in the statistical reports at the protein level when the changes in mRNA were zero. To investigate the overall correlation between these transcripts and DEPs, all identified mRNAs with DEPs were matched, followed by transformation of DEP and transcript volume ratios into log2 forms. An investigation of changes at both the transcript and protein levels revealed a weak correlation (Spearman correlation coefficient, R = 0.0840) for all genes and proteins assessed. We then calculated the correlation between the 382 DEPs and the 209 DEGs, and a positive correlation of R = 0.3006 was obtained when all significantly changed proteins with a cognate mRNA were considered (Fig 6). This result showed a modest correlation between the mRNA and protein levels (between the proteome and transcriptome), which was consistent with previously reported results [69], and accounted for the complexity of the gene expression regulation mechanism [54,70–72].

Forty-nine DEGs/DEPs were assigned GO terms to assess their functions that encompassed a vast range of cellular components, molecular functions and biological processes (S1 Fig). The GO analysis indicated that the DEGs/DEPs relevant to cellular, metabolic and biological regulation processes are possibly related to our study and showed that the DEGs/DEPs were relevant to molecular functions including catalysis, binding or structure.

Assignment to COG functional categories indicated that excluding carbohydrate transport and metabolism and the cytoskeleton, the DEGs/DEPs were classified into the categories of inorganic ion transport and metabolism and general function prediction.

**Fig 7:**
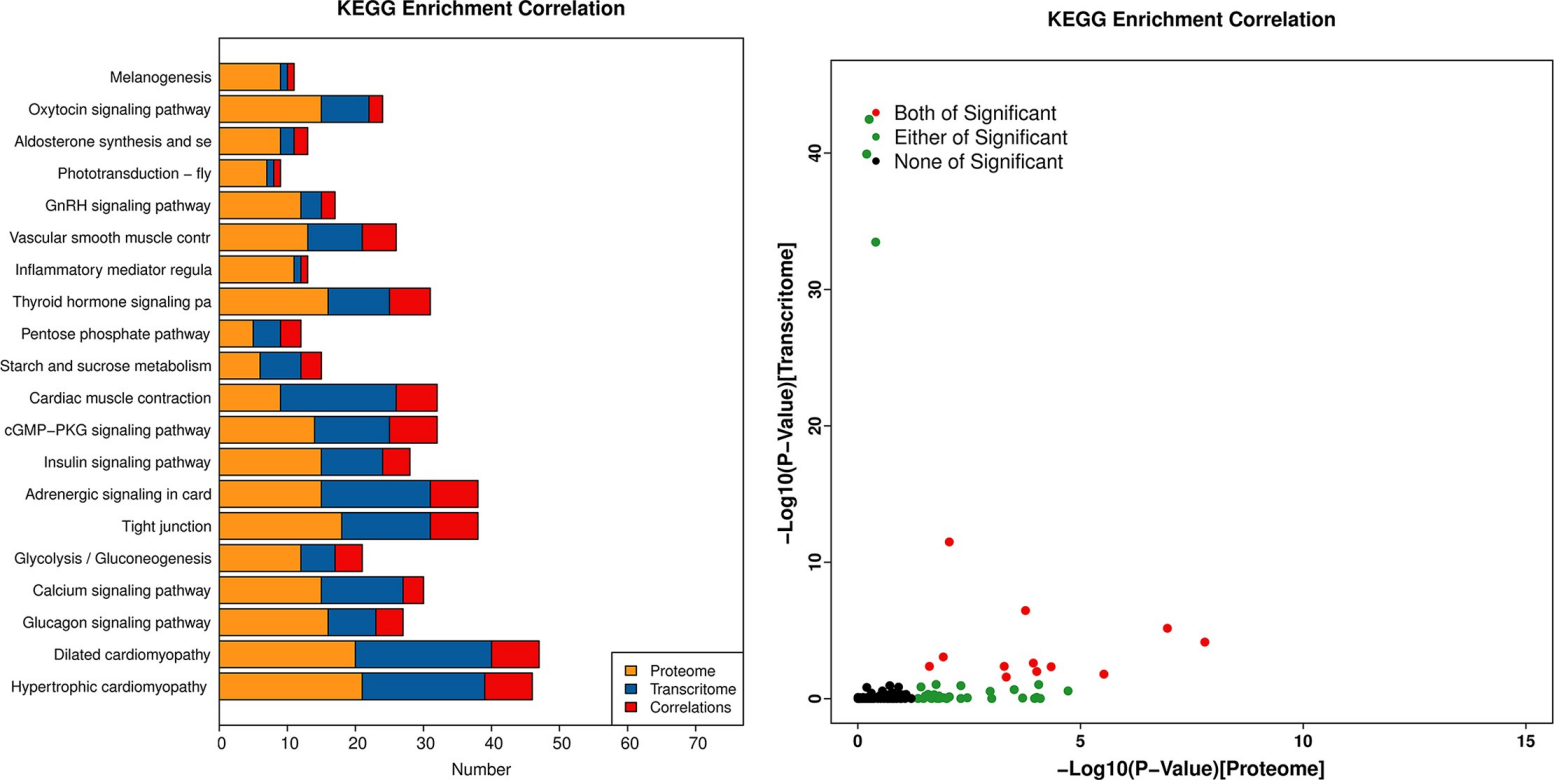
Correlation of KEGG enrichment between transcriptome and proteome. (A) Number of KEGG enrichment correlations between transcriptome and proteome. (B) The overview scatter diagram of KEGG enrichment correlations between the transcript levels and protein levels of genes.

Additionally, among the components of a single pathway the extent of comity between proteins and mRNA was studied. In total, 382 DEPs and 209 DEGs were mapped to 166 biological pathways, of which 12 pathways showed significant enrichment in both groups. This study involved the determination of vital DEPs and DEGs, involved in melanogenesis and the calcium, cGMP-PKG and adrenergic signaling in cardiomyocytes signaling pathway using pathway enrichment analysis (Fig 7).

## Discussion

Feathers have evolved to have diverse coloration that exhibits a wide spectrum of colors arranged in noticeable patterns. Either physical or chemical factors or a combination can form these colors. Melanin is the major factor responsible for the different types of colors described in the existing literature.

### DEGs and DEPs related to melanin synthesis

Pigmentation based on melanin is linked to a sequence of genes involved in the initiation and regulation of pigment production involving many signaling pathways as well as transcription factors such as the tyrosine kinase receptor *KIT* with *SCF* ligand, and *MITF* [73]. Many genes in the melanogenesis pathway are related to *MITF*, which is a sole transcription factor of the microphthalmia family that is involved in the regulation of melanocytes. *MITF* target genes regulate melanocyte pigmentation [74]. Previous studies revealed that *MITF* was involved in the development of melanocytes, along with reports of plumage color of Japanese quail and chicken and mutations in this gene [75].

**Fig 8:**
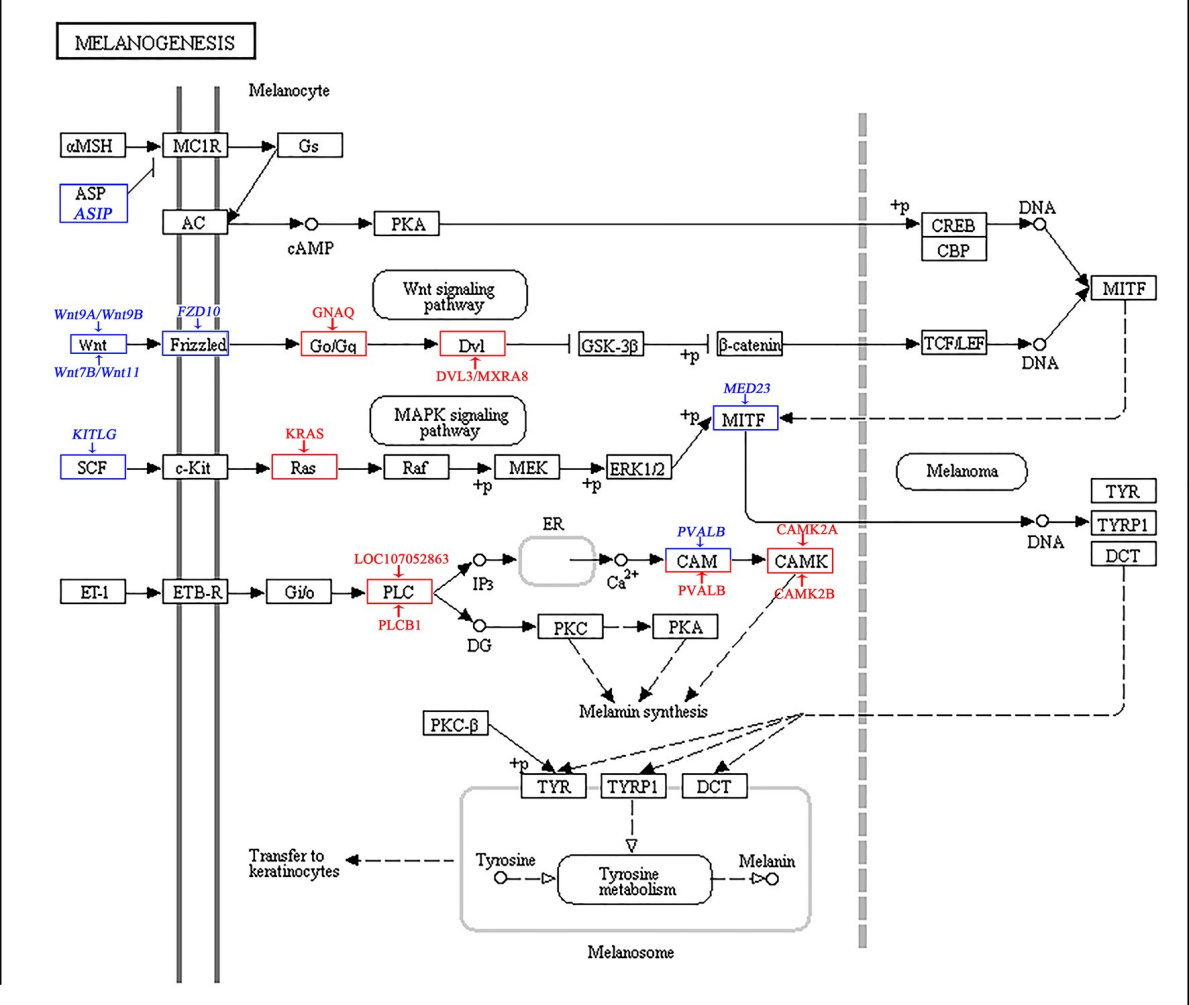
Differentially expressed feather color genes and proteins identified in the analyzed chicken feather follicles and their involvement in the melanogenesis pathway. DEGs have a blue frame, and the DEPs have a red frame.

In this study, a total of 8 DEGs (*ASIP*, *KITLG*, *FZD10*, *WNT7B*, *WNT9A*, *WNT9B*, *WNT11*, *PVALB*) and 9 DEPs (DVL3, KRAS, MXRA8, GNAQ, PVALB, CAMK2B, CAMK2A, PLCB1, LOC107052863) were found to be involved in the melanogenesis pathway (Fig 8). *MED23* was specially expressed and significantly upregulated in dorsal follicles of the neck. A new study revealed that zebrafish pigmentation is regulated by *MED23* via a modulation of the function of *MITF* as an enhancer [76]. GNAQ was significantly upregulated in the dorsal follicles of the neck in this pathway. Research showed that the *GNAQ* activates *MITF* via the MAPK pathway, and then affects melanin synthesis [77]. As a consequence, melanin synthesis can be regulated by influencing the expression of the *MITF* gene, which then regulates the development of feather coloration. In addition, we found that expression of the *MED23*, *FZD10*, *WNT7B* and *WNT11* genes peaked at approximately 8 weeks in the “Yufen I” H line, which is consistent with the molting cycle. The results are indicative of an extensive and vital role of these genes in melanin synthesis, which is consistent with previous research results. Therefore, we speculated that the above genes were related to the formation of Columbian plumage in the “Yufen I” H line.

### Pathways related to melanin synthesis

To further understand the DEGs and DEPs in the pathways that regulate chicken feather color development, we performed KEGG pathway analysis of DEGs and DEPs (p < 0.05). Correlation analysis and integration were performed on DEPs and DEGs annotated in the same pathway. Combining these results with those of the transcriptome and proteome analyses showed thatthe melanogenesis, adrenergic signaling in cardiomyocytes, calcium and cGMP-PKG signaling pathways were highly prevalent at the locations where different feather colors occurred in “Yufen I” H line feather follicles. Therefore, we speculate that these four pathways may be related to the Columbian plumage of the “Yufen I” H line, although we primarily focused on the melanogenesis pathway in this study. We found that the DEGs were mainly concentrated upstream of this pathway, while the DEPs were concentrated downstream of the pathway (Fig 8).

Interestingly, 5 proteins (PLCB1, LOC107052863, PVALB, CAMK2A, CAMK2B) and the *PAVLB* gene regulated melamine synthesis in the melanogenesis pathway. Few studies have previously reported on the correlation between melamine and pigmentation. The effect of melamine on the skin color of darkbarbel catfish showed that the factors necessary for melanin formation may be suppressed by intake of melamine, resulting in a significant reduction in melanin in the skin of the dorsal surface [78]. Therefore, we speculate that the 5 genes that regulate melamine synthesis may also be related to feather color deposition by affecting melanin synthesis.

## Conclusion

In summary, an incorporated and strong approach for analyzing the transcriptome and proteome was utilized to study the mechanism of melanin synthesis in feathers. Strikingly, this original report of a transcriptomics and proteomics analysis of the feather color in chicken follicles describes and reveals a set of DEGs that are putatively involved in determining feather color along with other physiological functions. *MED23*, *FZD10*, *WNT7B*, *WNT11* and GNAQ expression were significantly different in feather follicles with black and white stripes versus those with white feather color, and elucidating the functional roles of these genes in the regulation of feather color will be of particular interest in future studies. These results provide a potential understanding of the mechanism underlying Columbian plumage in the “Yufen I” H line and solid genetic resources that allow for the selection of birds with uniform plumage for breeding.

## Supporting information

S1 Fig GO enrichment correlation between transcriptome and proteome. (A) The number of GO enrichment correlations between the transcriptome and proteome. (B) Scatter diagram overview of GO enrichment correlation between the transcript and protein levels of genes.
(TIF)

S1 Table Information regarding the specific primers used for the qRT-PCR. (DOCX)

S2 Table The combined transcriptomic and proteomic analysis parameters. (DOCX)

S3 Table DEGs between the dorsal feather follicles and ventral feather follicles of the neck. (DOCX)

S4 Table DEPs between the dorsal feather follicles and ventral feather follicles of the neck. (DOCX)

S5 Table Correlation of DEPS and DEGs. (DOCX)
