# Supplementary figures and images for: Combined transcriptomics and proteomics forecast analysis for potential genes regulating the Columbian plumage color in chickens
Source: PLoS One. 2019 Nov 6;14(11):e0210850. doi: 10.1371/journal.pone.0210850 (PMC6834273; doi:10.1371/journal.pone.0210850)

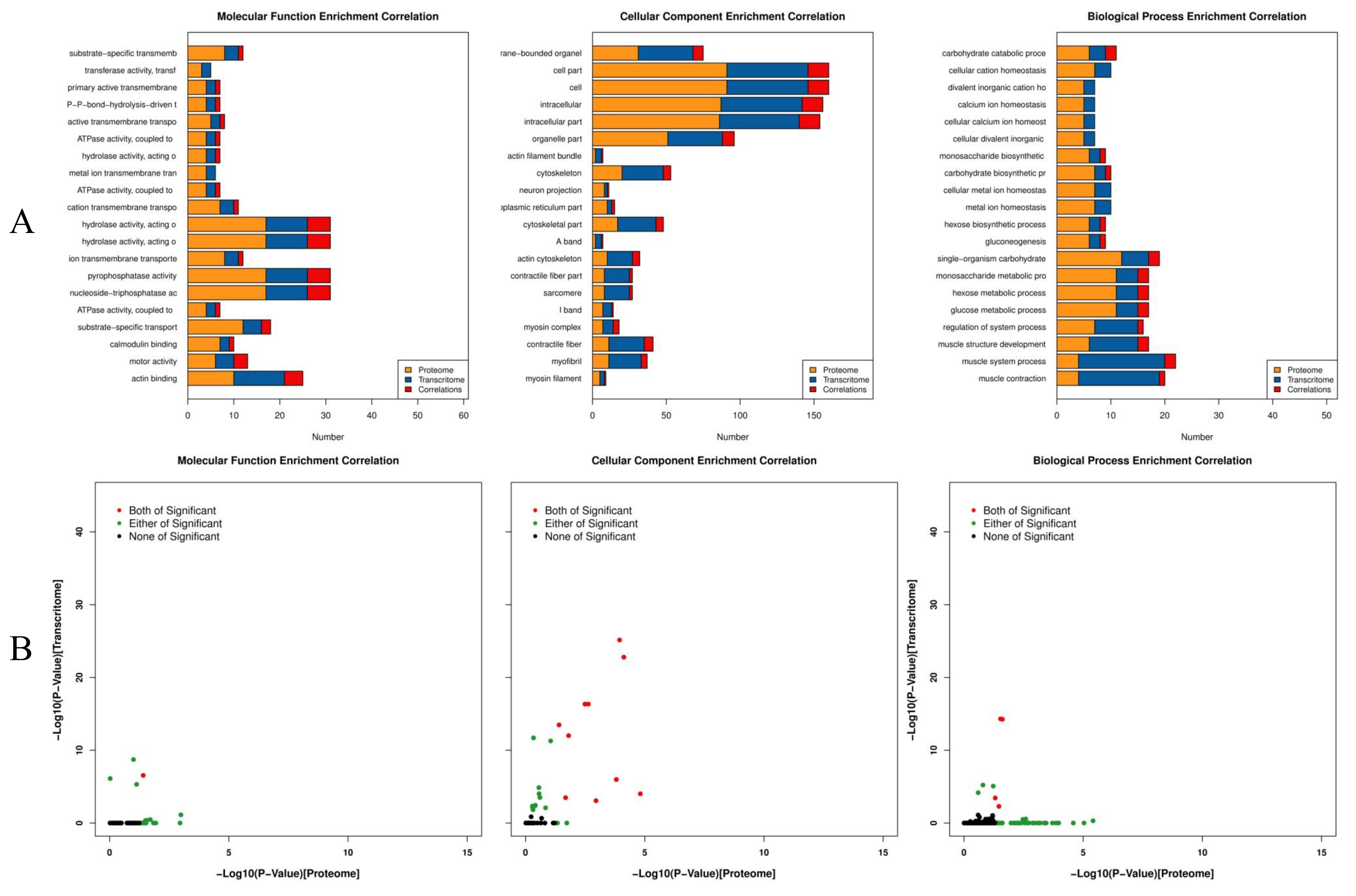

Supplement: S1 Fig — (A) The number of GO enrichment correlations between the transcriptome and proteome. (B) Scatter diagram overview of GO enrichment correlation between the transcript and protein levels of genes. (TIF) [file pone.0210850.s001.tif]
