# Supplementary material for: Combined transcriptomics and proteomics forecast analysis for potential genes regulating the Columbian plumage color in chickens
Source: PLoS One. 2019 Nov 6;14(11):e0210850. doi: 10.1371/journal.pone.0210850 (PMC6834273; doi:10.1371/journal.pone.0210850)
Supplement: S1 Table — (DOCX) [file pone.0210850.s002.docx]

**Supplementary Table 1.** Information regarding the specific primers used for the qRT-PCR

| **Gene** | **Forward primer** | **Reverse primer** | **Product Length(bp)** |
| --- | --- | --- | --- |
| PVALB | AGGAGAGATCCGAAAGTTGC | AAGCCGCTCCGGTCTTTATC | 196 |
| WNT11 | AGGTGAGACACCTGGACCTG | GGAGACAGGTCTTGAAAGCCTC | 180 |
| WNT9A | TGTGGGCATGAAGGTCATCAA | CGGGGGAAGGAGACATTTCC | 199 |
| WNT7B | TCGGAAGTGGATTTTCTACGTGT | CGAGTAGGAAGCAGGGAAGC | 297 |
| MED23 | TCGAGCTTTTCTACAGCGGTT | TGAGAGGTTGCTGTGGAACG | 290 |
| FZD10 | CTACACGAGTTTGCCCCCTT | CCCAACAACGGGTCAGATGA | 269 |
| KITLG | CGAAGATGGATAGCCTGGATTT | TCCAGAGTCGCTGTCACAAA | 202 |
