# Supplementary material for: Combined transcriptomics and proteomics forecast analysis for potential genes regulating the Columbian plumage color in chickens
Source: PLoS One. 2019 Nov 6;14(11):e0210850. doi: 10.1371/journal.pone.0210850 (PMC6834273; doi:10.1371/journal.pone.0210850)
Supplement: S2 Table — (DOCX) [file pone.0210850.s003.docx]

Supplementary Table 2. The combined analysis parameters of transcriptomic and proteomic.

| **Type** | **Value** |
| --- | --- |
| Protein_Unique Peptide | 1 |
| Protein_Fold Change | 1 |
| Protein_Significant | * |
| Gene_Fold Change | 2 |
| Gene_Significant | >0.8 |
| GO_Significant | <0.05 |
| Pathway_Significant | <0.05 |
| Blast_Identity | 100 |
| Blast_E value | 1.00E-08 |
| Top_number | 20 |
