# Supplementary material for: Combined transcriptomics and proteomics forecast analysis for potential genes regulating the Columbian plumage color in chickens
Source: PLoS One. 2019 Nov 6;14(11):e0210850. doi: 10.1371/journal.pone.0210850 (PMC6834273; doi:10.1371/journal.pone.0210850)
Supplement: S3 Table — (DOCX) [file pone.0210850.s004.docx]

**Supplementary Table 3.** Differentially expressed genes between the dorsal feather follicles of neck and the ventral feather follicles of neck.

| **GeneID** | **Symbol** | **B-Expression** | **A-Expression** | **log2Fold**  **Change** | **Up/Down** |
| --- | --- | --- | --- | --- | --- |
| 426927 | MED23 | 0 | 3.383 | 10.43831887 | Up |
| 100859398 | POLD2 | 0.046666667 | 8.483333333 | 7.506095019 | Up |
| 418451 | IGSF3 | 0.163333333 | 5.6 | 5.099535674 | Up |
| 107055094 | LOC107055094 | 5.016666667 | 57.14666667 | 3.509868363 | Up |
| 100859466 | LOC100859466 | 65.15333333 | 685.1233333 | 3.394452824 | Up |
| 107055270 | LOC107055270 | 105.8566667 | 1006.936667 | 3.249788909 | Up |
| 107057535 | LOC107057535 | 3.373333333 | 29.03 | 3.105297783 | Up |
| 107055265 | LOC107055265 | 152.48 | 1113.76 | 2.868746456 | Up |
| 101409259 | FK21 | 204.6166667 | 1246.61 | 2.607014624 | Up |
| 107055239 | LOC107055239 | 5.783333333 | 35.17 | 2.604372173 | Up |
| 107056779 | LOC107056779 | 13.82333333 | 79.91666667 | 2.531390863 | Up |
| 107055232 | LOC107055232 | 70.17666667 | 390.8666667 | 2.477613228 | Up |
| 101749907 | LOC101749907 | 20.21 | 105.11 | 2.378758705 | Up |
| 107055235 | LOC107055235 | 70.86333333 | 364.0266667 | 2.360932904 | Up |
| 107055253 | LOC107055253 | 70.86333333 | 364.0266667 | 2.360932904 | Up |
| 107055229 | LOC107055229 | 239.3366667 | 1215.71 | 2.344685782 | Up |
| 107056774 | LOC107056774 | 4.75 | 22.14 | 2.220655804 | Up |
| 107054169 | LOC107054169 | 85.89 | 392.4933333 | 2.192106073 | Up |
| 101409260 | FK27 | 344.1033333 | 1554.716667 | 2.175737913 | Up |
| 107054171 | LOC107054171 | 22.09 | 99.51666667 | 2.171544744 | Up |
| 107057577 | LOC107057577 | 85.7 | 376.4466667 | 2.135078375 | Up |
| 425854 | LOC425854 | 252.9366667 | 1060.223333 | 2.067520101 | Up |
| 107056781 | LOC107056781 | 151.8366667 | 623.4033333 | 2.037645643 | Up |
| 107049931 | LOC107049931 | 22.54333333 | 91.98 | 2.028619345 | Up |
| 107054158 | LOC107054158 | 25.59 | 104.2466667 | 2.026349201 | Up |
| 427937 | WNT7B | 0.516 | 2.013 | 2.010765421 | Up |
| 107056778 | LOC107056778 | 35.65666667 | 142.34 | 1.997097396 | Up |
| 107057561 | LOC107057561 | 255.31 | 1013.863333 | 1.989541242 | Up |
| 107050532 | LOC107050532 | 9.863333333 | 38.2 | 1.953425442 | Up |
| 107055236 | LOC107055236 | 140.4133333 | 538.9233333 | 1.940400114 | Up |
| 107050179 | LOC107050179 | 88.84666667 | 339.5333333 | 1.934163661 | Up |
| 107054165 | LOC107054165 | 73.01333333 | 268.2366667 | 1.877274609 | Up |
| 427060 | LOC427060 | 169.4033333 | 611.7666667 | 1.852519238 | Up |
| 107055228 | LOC107055228 | 219.6766667 | 787.57 | 1.842026518 | Up |
| 107055267 | LOC107055267 | 12.34 | 43.95 | 1.832520771 | Up |
| 107055109 | LOC107055109 | 69.52666667 | 235.7766667 | 1.761782622 | Up |
| 428287 | LOC428287 | 351.38 | 1188.103333 | 1.757556335 | Up |
| 771092 | WNT9B | 0.18 | 0.613 | 1.755275945 | Up |
| 107057555 | LOC107057555 | 149.0466667 | 501.8033333 | 1.751357943 | Up |
| 396028 | KITLG | 3.206 | 10.71 | 1.748355119 | Up |
| 107055237 | LOC107055237 | 87.19666667 | 290.9233333 | 1.738294121 | Up |
| 107055227 | LOC107055227 | 235.7966667 | 779.3233333 | 1.724678688 | Up |
| 107054166 | LOC107054166 | 244.63 | 795.61 | 1.701460071 | Up |
| 101751678 | SCFN | 32.41 | 105.28 | 1.699720468 | Up |
| 107055268 | LOC107055268 | 226.3666667 | 727.4466667 | 1.684179948 | Up |
| 107055269 | LOC107055269 | 227.48 | 729.2833333 | 1.680739714 | Up |
| 107055247 | LOC107055247 | 354.5033333 | 1136.096667 | 1.680214496 | Up |
| 425968 | EDMPN1 | 326.85 | 1035.346667 | 1.663413306 | Up |
| 107055231 | LOC107055231 | 130.11 | 407.5166667 | 1.647127215 | Up |
| 107057530 | LOC107057530 | 482.35 | 1502.206667 | 1.638931034 | Up |
| 771021 | LOC771021 | 390.67 | 1188.346667 | 1.604933385 | Up |
| 417474 | CLDN9 | 180.6933333 | 545.3733333 | 1.593700881 | Up |
| 107055233 | LOC107055233 | 354.7433333 | 1065.883333 | 1.587202059 | Up |
| 107049059 | LOC107049059 | 58.05 | 172.2233333 | 1.568912644 | Up |
| 770434 | LOC770434 | 14.45 | 42.06333333 | 1.541493689 | Up |
| 428291 | LOC428291 | 434.6333333 | 1263.566667 | 1.539631055 | Up |
| 431665 | SLC45A2 | 2.006 | 5.806 | 1.53503199 | Up |
| 107055120 | LOC107055120 | 541.6466667 | 1541.086667 | 1.50852405 | Up |
| 107049929 | LOC107049929 | 432.5866667 | 1215.91 | 1.490975343 | Up |
| 428295 | LOC428295 | 1060.773333 | 2975.66 | 1.48809328 | Up |
| 100859148 | LOC100859148 | 37.98333333 | 106.0066667 | 1.480716574 | Up |
| 428303 | LOC428303 | 206.5533333 | 575.4766667 | 1.478243091 | Up |
| 100858427 | LOC100858427 | 530.7833333 | 1454.076667 | 1.453908362 | Up |
| 431320 | LOC431320 | 54.21 | 147.0866667 | 1.440035561 | Up |
| 107055266 | LOC107055266 | 660.6566667 | 1786.416667 | 1.435095992 | Up |
| 107055108 | LOC107055108 | 626.1233333 | 1691.246667 | 1.433568319 | Up |
| 428299 | LOC428299 | 212.2866667 | 569.0666667 | 1.422583914 | Up |
| 428297 | LOC428297 | 283.08 | 758.5066667 | 1.421952036 | Up |
| 769926 | LOC769926 | 278.3233333 | 742.77 | 1.416153684 | Up |
| 107055262 | LOC107055262 | 205.03 | 542.8766667 | 1.404789456 | Up |
| 107049930 | LOC107049930 | 45.33666667 | 118.19 | 1.382357748 | Up |
| 428499 | CMBLL | 92.26 | 240.4666667 | 1.382059725 | Up |
| 100859427 | LOC100859427 | 1100.996667 | 2864.293333 | 1.379369146 | Up |
| 100859033 | LOC100859033 | 627.86 | 1618.146667 | 1.365827569 | Up |
| 107055225 | LOC107055225 | 525.6833333 | 1341.9 | 1.352011264 | Up |
| 107049053 | LOC107049053 | 78.23 | 199.3966667 | 1.349847422 | Up |
| 107055128 | EDMTF4 | 967.3133333 | 2421.866667 | 1.324064251 | Up |
| 107055248 | LOC107055248 | 2042.166667 | 5100.38 | 1.320504125 | Up |
| 107057527 | LOC107057527 | 589.3866667 | 1465.35 | 1.313958966 | Up |
| 107049038 | LOC107049038 | 491.22 | 1209.643333 | 1.300140521 | Up |
| 373885 | FZD10 | 7.773 | 17.836 | 1.200839367 | Up |
| 419147 | ASIP | 8.99 | 19.56 | 1.117580261 | Up |
| 395562 | WNT11 | 6.476 | 12.936 | 1.049633856 | Up |
| 396366 | TPM1 | 921.3166667 | 374.34 | -1.299347896 | Down |
| 404773 | MUSTN1 | 208.4933333 | 81.75333333 | -1.350651794 | Down |
| 395906 | DES | 1311.496667 | 487.75 | -1.427000363 | Down |
| 419607 | FHL3 | 174.08 | 62.30333333 | -1.482369205 | Down |
| 424691 | PGM1 | 58.26666667 | 20.84666667 | -1.48285406 | Down |
| BGI_novel_G000098 | - | 71.96333333 | 24.67333333 | -1.544309379 | Down |
| 107055281 | PDK2 | 34.27333333 | 11.25666667 | -1.606306833 | Down |
| 395260 | FLNC | 68.39 | 22.34333333 | -1.613940956 | Down |
| 396002 | AK1 | 97.48333333 | 30.32 | -1.68488583 | Down |
| 417124 | CUTA | 24.58 | 7.506666667 | -1.711240589 | Down |
| 426448 | SNTA1 | 34.27333333 | 10.46666667 | -1.711284453 | Down |
| 374069 | MYH1B | 31.70333333 | 9.423333333 | -1.750325153 | Down |
| 395552 | CCL1 | 23.32333333 | 6.823333333 | -1.773225389 | Down |
| 416925 | DGCR14 | 97.81333333 | 28.14 | -1.79740881 | Down |
| 396430 | TPM2 | 1783.956667 | 481.2 | -1.890372024 | Down |
| 107055091 | LOC107055091 | 56.79 | 15.28666667 | -1.893363058 | Down |
| 396468 | ATP1A2 | 75.16666667 | 20.15333333 | -1.899074549 | Down |
| 423756 | PITX3 | 18.17666667 | 4.77 | -1.930026483 | Down |
| 107049821 | SRPK3 | 34.77666667 | 8.89 | -1.967864333 | Down |
| BGI_novel_G000078 | - | 59.66 | 15.18 | -1.974592187 | Down |
| 429272 | MYO7L2 | 61.68666667 | 15.45333333 | -1.997040625 | Down |
| 395805 | MYOM1 | 15.95 | 3.983333333 | -2.001508307 | Down |
| 420391 | HHATL | 22.17 | 5.516666667 | -2.006740054 | Down |
| 100858920 | SYPL2 | 48.28 | 11.97666667 | -2.011199241 | Down |
| 395829 | WNT9A | 0.516 | 0.123 | -2.06443008 | Down |
| 770787 | FHL1 | 576.0866667 | 132.57 | -2.119531531 | Down |
| 418099 | MYBPC1 | 61.09333333 | 13.55666667 | -2.172012468 | Down |
| 396032 | TNNC1 | 247.2633333 | 54.55666667 | -2.180220913 | Down |
| 425201 | RTN4RL2 | 67.46 | 14.85333333 | -2.183245586 | Down |
| 423899 | NRAP | 18.71666667 | 4.036666667 | -2.213087158 | Down |
| 768936 | ALPK3 | 14.70666667 | 3.17 | -2.213915545 | Down |
| 107050916 | LOC107050916 | 114.8366667 | 24.59 | -2.223439719 | Down |
| 396269 | SRL | 19.58 | 4.186666667 | -2.225506801 | Down |
| 101748464 | RYR1L2 | 51.38666667 | 10.93 | -2.23310067 | Down |
| 107056413 | LOC107056413 | 14.27 | 3.003333333 | -2.248348824 | Down |
| 424552 | NEXN | 15.06666667 | 3.163333333 | -2.25184278 | Down |
| 395985 | CACNA1S | 19.49 | 4.073333333 | -2.258452306 | Down |
| 396034 | MYOM2 | 13.47 | 2.783333333 | -2.274864249 | Down |
| 424164 | KLHL41 | 20.52 | 4.24 | -2.274894561 | Down |
| 107055347 | LOC107055347 | 14.76 | 2.96 | -2.31802364 | Down |
| 107054910 | LOC107054910 | 22.75666667 | 4.53 | -2.328706296 | Down |
| 427177 | CMYA5 | 21.79 | 4.28 | -2.347983495 | Down |
| 107054910 | SLC25A4 | 22.75666667 | 4.53 | -2.328706296 | Down |
| 420208 | CA3A | 32.80333333 | 6.31 | -2.378130512 | Down |
| 396063 | SM1 | 130.5733333 | 25.07333333 | -2.380634578 | Down |
| 422546 | SLC25A4 | 185.4066667 | 35.43 | -2.387649749 | Down |
| 107050559 | LOC107050559 | 384.1166667 | 71.98666667 | -2.415742942 | Down |
| 374027 | NEB | 31.88 | 5.956666667 | -2.420074496 | Down |
| 772158 | LOC772158 | 84.47 | 15.67333333 | -2.430127013 | Down |
| 430280 | HSPB7 | 146.5833333 | 26.38666667 | -2.47384006 | Down |
| 414136 | HSPB2 | 15.63666667 | 2.786666667 | -2.488320653 | Down |
| 100857679 | CORO6 | 52.67333333 | 9.353333333 | -2.493520252 | Down |
| 107057560 | LOC107057560 | 241.9566667 | 40.63 | -2.574131421 | Down |
| 396089 | CRYAB | 35.90666667 | 5.94 | -2.595716893 | Down |
| 417310 | MYH1C | 107.1866667 | 17.71666667 | -2.596946359 | Down |
| 771780 | SMPX | 45.64666667 | 7.543333333 | -2.597235428 | Down |
| 374117 | JCHAIN | 431.5666667 | 71.17333333 | -2.600174728 | Down |
| 423743 | SYNPO2L | 11.87 | 1.91 | -2.635675392 | Down |
| 423610 | LDB3 | 53.32333333 | 8.56 | -2.639084267 | Down |
| 100857277 | SLN | 110.1566667 | 17.61 | -2.645089995 | Down |
| 427789 | MYH1G | 111.7533333 | 17.65 | -2.662577776 | Down |
| 396227 | HSPB1 | 574.5166667 | 89.6 | -2.680778109 | Down |
| 422682 | MYOZ2 | 98.44666667 | 14.75666667 | -2.737975486 | Down |
| 395279 | MYH7B | 80.99 | 12.12 | -2.740354088 | Down |
| 419915 | APOBEC2 | 152.0766667 | 22.66666667 | -2.746154665 | Down |
| 107053153 | TRIM54 | 20.61666667 | 3.063333333 | -2.750636829 | Down |
| 107054331 | CDH15 | 11.66666667 | 1.693333333 | -2.78445452 | Down |
| 395862 | PITX2 | 13.78666667 | 1.993333333 | -2.790018796 | Down |
| 395198 | CASQ2 | 44.46333333 | 6.393333333 | -2.797975889 | Down |
| 418426 | BTLA | 14.15 | 2 | -2.822730148 | Down |
| BGI_novel_G000097 | - | 917.32 | 129.63 | -2.823025458 | Down |
| 421534 | ACTA1 | 4510.053333 | 637.1966667 | -2.823333869 | Down |
| 374048 | MYOD1 | 23.59333333 | 3.236666667 | -2.865798561 | Down |
| 107054665 | LOC107054665 | 14.80333333 | 2.03 | -2.866370438 | Down |
| 100859605 | MPZ | 32.34333333 | 4.423333333 | -2.870262502 | Down |
| 426754 | TRIM63 | 30.69666667 | 4.166666667 | -2.881116409 | Down |
| 416299 | MYOT | 44.25333333 | 5.88 | -2.911898069 | Down |
| 416928 | IGLL1 | 10660.41 | 1386.886667 | -2.942341122 | Down |
| 417751 | LMOD2 | 23.55666667 | 3.06 | -2.94453185 | Down |
| 418741 | ADPRHL1 | 56.38 | 7.046666667 | -3.000170602 | Down |
| 101750657 | SMTNL1 | 20.73666667 | 2.543333333 | -3.027391544 | Down |
| 373896 | UCP3 | 15.56666667 | 1.89 | -3.04200191 | Down |
| 770260 | LOC770260 | 70.49 | 8.313333333 | -3.083919641 | Down |
| 396112 | RYR1 | 101.74 | 11.99333333 | -3.084582408 | Down |
| 396356 | MYLK2 | 40.58 | 4.706666667 | -3.107991277 | Down |
| BGI_novel_G000453 | - | 46.34 | 5.353333333 | -3.113748652 | Down |
| 107050875 | LOC107050875 | 23.06 | 2.646666667 | -3.123144101 | Down |
| 101749471 | LOC101749471 | 69.97 | 7.85 | -3.155971933 | Down |
| 419244 | EEF1A2 | 411.2333333 | 45.98666667 | -3.160669677 | Down |
| 417428 | CACNG1 | 42.09666667 | 4.706666667 | -3.160928413 | Down |
| 374064 | PFKM | 125.1466667 | 13.86 | -3.174620702 | Down |
| 417506 | MYL10 | 178.4766667 | 19.68333333 | -3.18068901 | Down |
| 421154 | MYBPH | 10.70666667 | 1.176666667 | -3.185731804 | Down |
| 107050974 | LOC107050974 | 66.57666667 | 7.086666667 | -3.231837544 | Down |
| 421161 | TNNI1 | 282.23 | 29.95666667 | -3.235922343 | Down |
| 107050927 | LOC107050927 | 108.5566667 | 11.43 | -3.247551019 | Down |
| 396507 | CKM | 1709.25 | 179.0933333 | -3.254579886 | Down |
| 396434 | TNNC2 | 982.9466667 | 101.65 | -3.273502925 | Down |
| 395761 | TNNT3 | 813.76 | 83.48333333 | -3.285043256 | Down |
| 396067 | MYL3 | 319.2766667 | 32.32 | -3.304308019 | Down |
| 395547 | TMOD4 | 42.11 | 4.2 | -3.325701643 | Down |
| 396470 | MYL1 | 1495.813333 | 148.77 | -3.329774612 | Down |
| 425457 | MYBPC2 | 42.18666667 | 4.153333333 | -3.344445531 | Down |
| 396009 | TNNT1 | 286.88 | 27.49 | -3.383468581 | Down |
| 396508 | CKMT2 | 27.41666667 | 2.596666667 | -3.400320445 | Down |
| 107051134 | LOC107051134 | 78.83333333 | 7.24 | -3.444744175 | Down |
| 107051274 | LOC107051274 | 4578.806667 | 419.69 | -3.447575657 | Down |
| 107049660 | LOC107049660 | 136.2466667 | 12.46 | -3.450844961 | Down |
| 378796 | CAV3 | 12.41 | 1.133333333 | -3.452858965 | Down |
| 422979 | CSRP3 | 44.04333333 | 3.986666667 | -3.465668772 | Down |
| 396528 | ATP2A1 | 486.8066667 | 43.99 | -3.468101419 | Down |
| 396386 | TNNI2 | 820.33 | 73.59666667 | -3.478492059 | Down |
| 107055299 | TCAP | 274.0066667 | 24.00333333 | -3.512904323 | Down |
| BGI_novel_G000392 | - | 9.813333333 | 0.84 | -3.546282033 | Down |
| 396459 | PVALB | 10.75333333 | 0.916666667 | -3.552242915 | Down |
| 423744 | MYOZ1 | 79.03333333 | 6.556666667 | -3.5914268 | Down |
| 417309 | MYH1A | 91.02 | 7.236666667 | -3.652786361 | Down |
| 418056 | MB | 199.4633333 | 15.64 | -3.672811147 | Down |
| 107050608 | LOC107050608 | 17.94 | 1.35 | -3.732148578 | Down |
| 107050715 | LOC107050715 | 108.43 | 7.963333333 | -3.767247713 | Down |
| 771119 | PRR33 | 31.18666667 | 2.196666667 | -3.827541491 | Down |
| BGI_novel_G000569 | - | 39.47666667 | 2.773333333 | -3.831307244 | Down |
| 373960 | SMYD1 | 26.55666667 | 1.803333333 | -3.880336076 | Down |
| 100859189 | MZB1 | 47.16666667 | 2.773333333 | -4.088074714 | Down |
| 416874 | MYL2 | 30.37333333 | 1.716666667 | -4.14512341 | Down |
| 107057587 | ARX | 7.96 | 0.44 | -4.177193002 | Down |
