# Supplementary material for: Combined transcriptomics and proteomics forecast analysis for potential genes regulating the Columbian plumage color in chickens
Source: PLoS One. 2019 Nov 6;14(11):e0210850. doi: 10.1371/journal.pone.0210850 (PMC6834273; doi:10.1371/journal.pone.0210850)
Supplement: S4 Table — (DOCX) [file pone.0210850.s005.docx]

**Supplementary Table 4.** Differentially expressed proteins between the dorsal feather follicles of neck and the ventral feather follicles of neck.

| **No.** | **Protein_ID** | **Description** | ***P* value** | **NCBInr Accession** | **UP/**  **DOWM** |
| --- | --- | --- | --- | --- | --- |
| 1 | NP_001091708.1 | THO complex subunit 7 homolog [Gallus gallus] | 0.003149 | gi\|148277631\|ref\|NP_001091708.1\| | UP |
| 2 | XP_015142622.1 | PREDICTED MAX gene-associated protein isoform X9 [Gallus gallus] | 2.42E-05 | gi\|363734424\|ref\|XP_421144.3\| | UP |
| 3 | XP_015138084.1 | PREDICTED desmocollin-2 isoform X4 [Gallus gallus] | 0.01732 | gi\|118086928\|ref\|XP_426081.2\| | UP |
| 4 | XP_004949436.1 | PREDICTED fibrillin-2 isoform X2 [Gallus gallus] | 0.02258 | gi\|363744700\|ref\|XP_424715.3\| | UP |
| 5 | NP_989456.1 | sulfhydryl oxidase 1 precursor [Gallus gallus] | 0.01233 | gi\|45383856\|ref\|NP_989456.1\| | UP |
| 6 | XP_421513.1 | PREDICTED uncharacterized protein LOC423629 [Gallus gallus] | 0.04264 | gi\|50749164\|ref\|XP_421513.1\| | UP |
| 7 | XP_015155433.1 | PREDICTED methyl-CpG-binding domain protein 3 isoform X2 [Gallus gallus] | 0.01772 | gi\|395513241\|ref\|XP_003760836.1\| | UP |
| 8 | NP_998744.1 | GTP-binding protein GEM [Gallus gallus] | 0.005206 | gi\|47087177\|ref\|NP_998744.1\| | UP |
| 9 | BGI_novel_T008562 | gene=417127 [translate_table standard] | 0.02418 | - | UP |
| 10 | BGI_novel_T003269 | gene=100857197 [translate_table standard] | 0.0158 | - | UP |
| 11 | NP_001026134.1 | Golgi reassembly-stacking protein 1 [Gallus gallus] | 0.01173 | gi\|71896608\|ref\|NP_001026134.1\| | UP |
| 12 | XP_015146987.1 | PREDICTED prothymosin alpha [Gallus gallus] | 0.004395 | - | UP |
| 13 | NP_001182406.1 | PREDICTED keratin-like protein KRT222 isoform X1 [Gallus gallus] | 0.003659 | gi\|306518593\|ref\|NP_001182406.1\| | UP |
| 14 | XP_015139758.1 | PREDICTED protein phosphatase 1 regulatory subunit 14C isoform X2 [Gallus gallus] | 0.004843 | gi\|19311014\|ref\|NP_597844.1\| | UP |
| 15 | XP_015142608.1 | PREDICTED cytosolic phospholipase A2 beta isoform X2 [Gallus gallus] | 0.0179 | gi\|326920549\|ref\|XP_003206533.1\| | UP |
| 16 | XP_422682.1 | PREDICTED dnaJ homolog subfamily B member 11 [Gallus gallus] | 0.0005039 | gi\|50752156\|ref\|XP_422682.1\| | UP |
| 17 | XP_015145186.1 | PREDICTED coiled-coil domain-containing protein 173 [Gallus gallus] | 0.007719 | gi\|363736212\|ref\|XP_422007.3\| | UP |
| 18 | XP_015133914.1 | PREDICTED glycerophosphoinositol inositolphosphodiesterase GDPD2 [Gallus gallus] | 0.02686 | gi\|363732986\|ref\|XP_420208.3\| | UP |
| 19 | NP_001025739.2 | lysophosphatidylcholine acyltransferase 2 [Gallus gallus] | 0.01099 | gi\|347800751\|ref\|NP_001025739.2\| | UP |
| 20 | XP_417845.2 | PREDICTED UDP-N-acetylglucosamine--dolichyl-phosphate N-acetylglucosaminephosphotransferase isoform X2 [Gallus gallus] | 0.00125 | gi\|118101814\|ref\|XP_417845.2\| | UP |
| 21 | BGI_novel_T008952 | gene=422124 [translate_table standard] | 0.000753 | - | UP |
| 22 | XP_001234653.3 | PREDICTED protein FAM8A1 [Gallus gallus] | 0.002945 | gi\|449270052\|gb\|EMC80776.1\| | UP |
| 23 | XP_015148166.1 | PREDICTED VPS9 domain-containing protein 1 [Gallus gallus] | 0.001243 | gi\|363738402\|ref\|XP_414200.3\| | UP |
| 24 | XP_015148307.1 | PREDICTED vacuolar fusion protein MON1 homolog A isoform X1 [Gallus gallus] | 0.009305 | gi\|363738460\|ref\|XP_003642014.1\| | UP |
| 25 | XP_015151601.1 | PREDICTED inactive serine/threonine-protein kinase TEX14 isoform X6 [Gallus gallus] | 0.0008977 | gi\|326931483\|ref\|XP_003211858.1\| | UP |
| 26 | XP_015136581.1 | PREDICTED cerebral cavernous malformations 2 protein isoform X1 [Gallus gallus] | 0.00962 | gi\|326921440\|ref\|XP_003206967.1\| | UP |
| 27 | XP_015146605.1 | PREDICTED ribonucleoprotein PTB-binding 2 isoform X1 [Gallus gallus] | 0.02443 | gi\|363736681\|ref\|XP_422525.3\| | UP |
| 28 | XP_015141734.1 | PREDICTED neuron navigator 2 isoform X12 [Gallus gallus] | 0.01141 | gi\|326919775\|ref\|XP_003206153.1\| | UP |
| 29 | NP_001025565.1 | high mobility group protein 20A [Gallus gallus] | 0.01652 | gi\|71896259\|ref\|NP_001025565.1\| | UP |
| 30 | XP_416800.2 | PREDICTED peroxiredoxin-4 isoform X2 [Gallus gallus] | 0.004239 | gi\|118084001\|ref\|XP_416800.2\| | UP |
| 31 | NP_001264300.1 | ribosomal RNA-processing protein 7 homolog A [Gallus gallus] | 0.001857 | gi\|363727780\|ref\|XP_416213.3\| | UP |
| 32 | XP_015128300.1 | PREDICTED phosphorylase b kinase regulatory subunit alpha, liver isoform isoform X3 [Gallus gallus] | 0.008623 | gi\|363728840\|ref\|XP_416811.3\| | UP |
| 33 | XP_415503.2 | PREDICTED ubiquinone biosynthesis protein COQ4 homolog, mitochondrial [Gallus gallus] | 0.01886 | gi\|118099263\|ref\|XP_415503.2\| | UP |
| 34 | XP_015150483.1 | PREDICTED LOW QUALITY PROTEIN tenascin-X [Gallus gallus] | 0.0276 | gi\|148356986\|dbj\|BAF63010.1\| | UP |
| 35 | XP_015135460.1 | PREDICTED probable helicase with zinc finger domain isoform X4 [Gallus gallus] | 0.001796 | gi\|118099746\|ref\|XP_415679.2\| | UP |
| 36 | XP_004943451.1 | PREDICTED segment polarity protein dishevelled homolog DVL-3 isoform X6 [Gallus gallus] | 0.0005105 | gi\|183986681\|ref\|NP_001116929.1\| | UP |
| 37 | XP_424060.5 | PREDICTED lysyl oxidase homolog 1 [Gallus gallus] | 0.001771 | gi\|363737533\|ref\|XP_424060.3\| | UP |
| 38 | NP_001264470.1 | protein FAM192A [Gallus gallus] | 0.0188 | gi\|449268841\|gb\|EMC79678.1\| | UP |
| 39 | XP_015154054.1 | PREDICTED tropomyosin alpha-3 chain isoform X4 [Gallus gallus] | 0.03782 | gi\|350537089\|ref\|NP_001232856.1\| | UP |
| 40 | NP_001006353.1 | coiled-coil domain-containing protein 12 [Gallus gallus] | 0.004393 | gi\|57530671\|ref\|NP_001006353.1\| | UP |
| 41 | BGI_novel_T003787 | gene=426948 [translate_table standard] | 0.013 | - | UP |
| 42 | NP_989590.1 | prostaglandin-H2 D-isomerase precursor [Gallus gallus] | 0.04083 | gi\|45383612\|ref\|NP_989590.1\| | UP |
| 43 | BGI_novel_T000666 | gene=418383 [translate_table standard] | 6.89E-05 | - | UP |
| 44 | XP_015151309.1 | PREDICTED inositol polyphosphate 5-phosphatase K isoform X1 [Gallus gallus] | 0.01826 | gi\|363741105\|ref\|XP_001234813.2\| | UP |
| 45 | XP_015149312.1 | PREDICTED parathymosin [Gallus gallus] | 0.0009623 | - | UP |
| 46 | NP_990606.1 | metallothionein [Gallus gallus] | 0.01065 | gi\|380865607\|gb\|AFF19511.1\| | UP |
| 47 | NP_990298.1 | fibromodulin precursor [Gallus gallus] | 0.02904 | gi\|45384456\|ref\|NP_990298.1\| | UP |
| 48 | XP_015151961.1 | PREDICTED p53 and DNA damage-regulated protein 1 [Gallus gallus] | 0.01397 | gi\|350536327\|ref\|NP_001232708.1\| | UP |
| 49 | NP_001073229.1 | BRCA1-associated protein [Gallus gallus] | 0.02775 | gi\|119331156\|ref\|NP_001073229.1\| | UP |
| 50 | XP_415193.5 | PREDICTED ankyrin repeat domain-containing protein 13A isoform X2 [Gallus gallus] | 0.0002747 | gi\|449279225\|gb\|EMC86860.1\| | UP |
| 51 | XP_015145518.1 | PREDICTED band 4.1-like protein 5 isoform X3 [Gallus gallus] | 0.02432 | gi\|363736309\|ref\|XP_422083.3\| | UP |
| 52 | XP_015139238.1 | PREDICTED regulator of microtubule dynamics protein 2 [Gallus gallus] | 0.01611 | gi\|363731570\|ref\|XP_419514.3\| | UP |
| 53 | XP_015145728.1 | PREDICTED pre-B-cell leukemia transcription factor 1 isoform X2 [Gallus gallus] | 0.009468 | gi\|402858031\|ref\|XP_003893534.1\| | UP |
| 54 | NP_990604.1 | nuclear factor 1 A-type [Gallus gallus] | 0.04474 | gi\|45384030\|ref\|NP_990604.1\| | UP |
| 55 | NP_001186613.1 | RNA-binding protein 25 [Gallus gallus] | 0.007545 | gi\|314122351\|ref\|NP_001186613.1\| | UP |
| 56 | NP_001264791.1 | U6 snRNA-associated Sm-like protein LSm3 [Gallus gallus] | 0.000715 | gi\|350536047\|ref\|NP_001232226.1\| | UP |
| 57 | XP_015128820.1 | PREDICTED ranBP-type and C3HC4-type zinc finger-containing protein 1-like isoform X3 [Gallus gallus] | 0.0477 | gi\|465950745\|gb\|EMP23949.1\| | UP |
| 58 | XP_015153236.1 | PREDICTED serine/arginine-rich splicing factor 4 isoform X1 [Gallus gallus] | 0.003014 | gi\|326932930\|ref\|XP_003212564.1\| | UP |
| 59 | XP_003641978.2 | PREDICTED uncharacterized protein LOC776594 [Gallus gallus] | 0.04009 | gi\|326927487\|ref\|XP_003209924.1\| | UP |
| 60 | XP_015134776.1 | PREDICTED spectrin alpha chain, non-erythrocytic 1 isoform X9 [Gallus gallus] | 0.007284 | gi\|449477910\|ref\|XP_004174387.1\| | UP |
| 61 | XP_015136871.1 | PREDICTED tRNA (cytosine(38)-C(5))-methyltransferase isoform X1 [Gallus gallus] | 0.0009166 | gi\|67514595\|ref\|NP_001020002.1\| | UP |
| 62 | BGI_novel_T006597 | gene=374270 [translate_table standard] | 0.04951 | gi\|98162705\|ref\|NP_989683.3\| | UP |
| 63 | XP_015133994.1 | PREDICTED protein POF1B [Gallus gallus] | 0.02952 | gi\|363732609\|ref\|XP_420252.3\| | UP |
| 64 | XP_015131273.1 | PREDICTED cAMP-dependent protein kinase type II-beta regulatory subunit isoform X1 [Gallus gallus] | 0.008105 | gi\|327273640\|ref\|XP_003221588.1\| | UP |
| 65 | XP_015145880.1 | PREDICTED E-selectin isoform X6 [Gallus gallus] | 0.0006427 | gi\|363736461\|ref\|XP_422207.3\| | UP |
| 66 | XP_015130487.1 | PREDICTED NEDD8-conjugating enzyme Ubc12-like [Gallus gallus] | 0.0005289 | gi\|326935982\|ref\|XP_003214041.1\| | UP |
| 67 | NP_990246.1 | eyes absent homolog 2 [Gallus gallus] | 0.0008195 | gi\|45382509\|ref\|NP_990246.1\| | UP |
| 68 | XP_015147981.1 | PREDICTED pyruvate dehydrogenase [acetyl-transferring]-phosphatase 2, mitochondrial [Gallus gallus] | 0.02704 | gi\|363738181\|ref\|XP_425122.3\| | UP |
| 69 | XP_004940907.1 | PREDICTED E3 ubiquitin-protein ligase RNF128 isoform X2 [Gallus gallus] | 0.005512 | gi\|326924632\|ref\|XP_003208529.1\| | UP |
| 70 | XP_426718.4 | PREDICTED rho guanine nucleotide exchange factor 26 [Gallus gallus] | 0.01318 | gi\|363737437\|ref\|XP_426718.3\| | UP |
| 71 | XP_015139023.1 | PREDICTED 1-phosphatidylinositol 4,5-bisphosphate phosphodiesterase beta-1-like [Gallus gallus] | 0.04754 | gi\|426390935\|ref\|XP_004061847.1\| | UP |
| 72 | XP_015151470.1 | PREDICTED derlin-2 [Gallus gallus] | 0.01649 | gi\|118100103\|ref\|XP_415746.2\| | UP |
| 73 | NP_001153187.1 | ER lumen protein-retaining receptor 2 [Gallus gallus] | 0.0003885 | gi\|228480244\|ref\|NP_001153187.1\| | UP |
| 74 | XP_003643883.2 | PREDICTED LOW QUALITY PROTEIN cyclic AMP-dependent transcription factor ATF-7 [Gallus gallus] | 0.01457 | gi\|363747004\|ref\|XP_003643883.1\| | UP |
| 75 | XP_015153812.1 | PREDICTED acidic leucine-rich nuclear phosphoprotein 32 family member E isoform X1 [Gallus gallus] | 0.01511 | gi\|53133656\|emb\|CAG32157.1\| | UP |
| 76 | XP_015143523.1 | PREDICTED poly(ADP-ribose) glycohydrolase isoform X4 [Gallus gallus] | 6.05E-06 | gi\|118092423\|ref\|XP_421502.2\| | UP |
| 77 | XP_015128406.1 | PREDICTED epoxide hydrolase 3-like [Gallus gallus] | 0.03632 | gi\|363736677\|ref\|XP_422345.3\| | UP |
| 78 | XP_015128705.1 | PREDICTED LOW QUALITY PROTEIN WD repeat-containing protein 13 [Gallus gallus] | 0.002254 | gi\|327263802\|ref\|XP_003216706.1\| | UP |
| 79 | NP_001264663.1 | dynein light chain Tctex-type 3 [Gallus gallus] | 0.03056 | gi\|363728802\|ref\|XP_416782.3\| | UP |
| 80 | BGI_novel_T001185 | gene=428091 [translate_table standard] | 0.04498 | gi\|363729227\|ref\|XP_425649.3\| | UP |
| 81 | NP_001184165.1 | angiopoietin-related protein 5 precursor [Gallus gallus] | 0.00511 | gi\|308736956\|ref\|NP_001184165.1\| | UP |
| 82 | XP_015136730.1 | PREDICTED interleukin-1 receptor-associated kinase 4 isoform X1 [Gallus gallus] | 0.02544 | gi\|326911443\|ref\|XP_003202068.1\| | UP |
| 83 | XP_001232670.1 | PREDICTED UBX domain-containing protein 6 isoform X2 [Gallus gallus] | 0.002719 | gi\|118103170\|ref\|XP_001232670.1\| | UP |
| 84 | BGI_novel_T000423 | gene=418163 [translate_table standard] | 0.001391 | gi\|50729026\|ref\|XP_416394.1\| | UP |
| 85 | XP_015149082.1 | PREDICTED F-box/WD repeat-containing protein 11 isoform X4 [Gallus gallus] | 0.03559 | gi\|86129432\|ref\|NP_001034351.1\| | UP |
| 86 | XP_004940414.1 | PREDICTED single-minded homolog 1 isoform X2 [Gallus gallus] | 0.03149 | gi\|118088683\|ref\|XP_419817.2\| | UP |
| 87 | XP_418830.2 | PREDICTED mammalian ependymin-related protein 1 [Gallus gallus] | 0.01404 | gi\|118086182\|ref\|XP_418830.2\| | UP |
| 88 | NP_001243091.1 | PREDICTED GTPase KRas isoform X2 [Gallus gallus] | 0.0007262 | gi\|371872727\|ref\|NP_001243091.1\| | UP |
| 89 | XP_004940666.1 | PREDICTED RNA-binding protein 41 isoform X2 [Gallus gallus] | 0.003726 | gi\|363732706\|ref\|XP_426256.3\| | UP |
| 90 | XP_015139617.1 | PREDICTED rho GTPase-activating protein 18 isoform X2 [Gallus gallus] | 0.01384 | gi\|363732095\|ref\|XP_419743.3\| | UP |
| 91 | XP_015156940.1 | PREDICTED Down syndrome critical region protein 3 isoform X2 [Gallus gallus] | 0.001022 | gi\|50729967\|ref\|XP_416728.1\| | UP |
| 92 | XP_001231868.2 | PREDICTED platelet-derived growth factor receptor-like protein isoform X3 [Gallus gallus] | 0.004001 | gi\|363733691\|ref\|XP_001231868.2\| | UP |
| 93 | XP_015147682.1 | PREDICTED pro-cathepsin H [Gallus gallus] | 0.04536 | gi\|330376140\|gb\|AEC13302.1\| | UP |
| 94 | NP_001025740.1 | HEAT repeat-containing protein 3 [Gallus gallus] | 0.004567 | gi\|71895555\|ref\|NP_001025740.1\| | UP |
| 95 | BGI_novel_T003979 | gene=374256 [translate_table standard] | 0.002343 | - | UP |
| 96 | XP_015151963.1 | PREDICTED BPI fold-containing family B member 4 [Gallus gallus] | 0.0004024 | gi\|363741533\|ref\|XP_425718.3\| | UP |
| 97 | XP_417757.1 | PREDICTED yrdC domain-containing protein, mitochondrial isoform X2 [Gallus gallus] | 6.35E-05 | gi\|50759736\|ref\|XP_417757.1\| | UP |
| 98 | XP_003643122.3 | PREDICTED transmembrane emp24 domain-containing protein 7 [Gallus gallus] | 0.01656 | gi\|363744779\|ref\|XP_003643122.1\| | UP |
| 99 | NP_001013014.1 | ER lumen protein-retaining receptor 3 [Gallus gallus] | 3.98E-05 | gi\|61563734\|ref\|NP_001013014.1\| | UP |
| 100 | NP_001265074.1 | 1-acylglycerol-3-phosphate O-acyltransferase ABHD5 [Gallus gallus] | 0.0007369 | gi\|186702998\|gb\|ACC91733.1\| | UP |
| 101 | XP_015130105.1 | PREDICTED astrocytic phosphoprotein PEA-15-like [Gallus gallus] | 0.001431 | gi\|449273958\|gb\|EMC83285.1\| | UP |
| 102 | XP_015146821.1 | PREDICTED armadillo repeat-containing protein 8 isoform X7 [Gallus gallus] | 0.02495 | gi\|356461036\|ref\|NP_001239092.1\| | UP |
| 103 | XP_015130080.1 | PREDICTED translation initiation factor IF-2-like, partial [Gallus gallus] | 0.04168 | gi\|465956437\|gb\|EMP26944.1\| | UP |
| 104 | XP_015137628.1 | PREDICTED disco-interacting protein 2 homolog C [Gallus gallus] | 0.0342 | gi\|149634700\|ref\|XP_001509877.1\| | UP |
| 105 | NP_001264337.1 | fetuin-B precursor [Gallus gallus] | 0.01662 | gi\|50752383\|ref\|XP_422765.1\| | UP |
| 106 | XP_015153661.1 | PREDICTED ubiquitin-associated and SH3 domain-containing protein B [Gallus gallus] | 0.03752 | gi\|449489238\|ref\|XP_004176736.1\| | UP |
| 107 | XP_015139393.1 | PREDICTED WD repeat-containing protein 26 [Gallus gallus] | 0.04972 | gi\|363731477\|ref\|XP_419389.3\| | UP |
| 108 | XP_423078.3 | PREDICTED kin of IRRE-like protein 1 [Gallus gallus] | 0.03286 | gi\|363742661\|ref\|XP_423078.3\| | UP |
| 109 | BGI_novel_T008465 | gene=417202 [translate_table standard] | 0.002203 | - | UP |
| 110 | NP_001007901.1 | PREDICTED protein yippee-like 5 isoform X1 [Gallus gallus] | 0.0012 | gi\|350582577\|ref\|XP_003481305.1\| | UP |
| 111 | NP_990598.1 | nucleophosmin [Gallus gallus] | 3.73E-05 | gi\|45383996\|ref\|NP_990598.1\| | UP |
| 112 | NP_989996.1 | tumor necrosis factor receptor superfamily member 5 precursor [Gallus gallus] | 0.02902 | gi\|45382793\|ref\|NP_989996.1\| | UP |
| 113 | XP_424268.3 | PREDICTED protein phosphatase 1 regulatory subunit 1B isoform X2 [Gallus gallus] | 2.38E-05 | gi\|363743510\|ref\|XP_424268.3\| | UP |
| 114 | XP_015128680.1 | PREDICTED caseinolytic peptidase B protein homolog [Gallus gallus] | 5.48E-05 | gi\|449485970\|ref\|XP_002187730.2\| | UP |
| 115 | NP_001075176.1 | cAMP-regulated phosphoprotein 19 [Gallus gallus] | 0.0194 | gi\|126165246\|ref\|NP_001075176.1\| | UP |
| 116 | XP_015128188.1 | PREDICTED stomatin-like protein 2, mitochondrial [Gallus gallus] | 0.04763 | gi\|449514127\|ref\|XP_002190090.2\| | UP |
| 117 | NP_001006342.1 | myeloid-derived growth factor precursor [Gallus gallus] | 0.004199 | gi\|57530610\|ref\|NP_001006342.1\| | UP |
| 118 | BGI_novel_T004554 | gene=BGI_novel_G000045 [translate_table standard] | 0.0114 | gi\|363735185\|ref\|XP_001231398.2\| | UP |
| 119 | XP_418537.1 | PREDICTED xylulose kinase isoform X2 [Gallus gallus] | 0.01257 | gi\|50732227\|ref\|XP_418537.1\| | UP |
| 120 | XP_420136.2 | PREDICTED peptidyl-prolyl cis-trans isomerase NIMA-interacting 4 [Gallus gallus] | 0.002683 | gi\|118089325\|ref\|XP_420136.2\| | UP |
| 121 | XP_415461.1 | PREDICTED allograft inflammatory factor 1-like [Gallus gallus] | 0.01175 | gi\|50757279\|ref\|XP_415461.1\| | UP |
| 122 | XP_001234988.1 | PREDICTED E3 ubiquitin-protein ligase pellino homolog 2 [Gallus gallus] | 0.00576 | gi\|118092175\|ref\|XP_001234988.1\| | UP |
| 123 | XP_015149512.1 | PREDICTED prolyl 4-hydroxylase subunit alpha-2 isoform X5 [Gallus gallus] | 0.002594 | gi\|54792285\|emb\|CAG28668.1\| | UP |
| 124 | XP_015144624.1 | PREDICTED collagen alpha-1(III) chain isoform X1 [Gallus gallus] | 0.0118 | gi\|211572\|gb\|AAA18519.1\| | UP |
| 125 | XP_015149981.1 | PREDICTED ubiquitin domain-containing protein UBFD1 [Gallus gallus] | 0.03657 | gi\|363739413\|ref\|XP_001234543.2\| | UP |
| 126 | NP_001004389.2 | T-complex protein 1 subunit theta [Gallus gallus] | 6.35E-05 | gi\|52138673\|ref\|NP_001004389.1\| | UP |
| 127 | XP_015136072.1 | PREDICTED versican core protein isoform X2 [Gallus gallus] | 0.04712 | gi\|46048882\|ref\|NP_990118.1\| | UP |
| 128 | NP_001231834.1 | transcription initiation factor TFIID subunit 11 [Gallus gallus] | 0.008409 | gi\|349732112\|ref\|NP_001231834.1\| | UP |
| 129 | XP_004949571.1 | PREDICTED uncharacterized protein KIAA1958 homolog isoform X2 [Gallus gallus] | 0.0007598 | gi\|118104448\|ref\|XP_429189.2\| | UP |
| 130 | XP_015144013.1 | PREDICTED PDZ and LIM domain protein 1 isoform X2 [Gallus gallus] | 0.008193 | gi\|363735661\|ref\|XP_426503.3\| | UP |
| 131 | XP_015142233.1 | PREDICTED mucin-5AC [Gallus gallus] | 0.02334 | gi\|363734286\|ref\|XP_421033.3\| | UP |
| 132 | XP_015145444.1 | PREDICTED TGF-beta receptor type-2 [Gallus gallus] | 0.03066 | gi\|50750712\|ref\|XP_422108.1\| | UP |
| 133 | XP_015131374.1 | PREDICTED thioredoxin domain-containing protein 5 isoform X1 [Gallus gallus] | 3.15E-05 | gi\|57530789\|ref\|NP_001006374.1\| | UP |
| 134 | XP_015154191.1 | PREDICTED mitochondrial import inner membrane translocase subunit Tim17-A isoform X2 [Gallus gallus] | 0.03597 | gi\|71895925\|ref\|NP_001026197.1\| | UP |
| 135 | NP_990605.2 | myosin-11 [Gallus gallus] | 0.02185 | gi\|3915778\|sp\|P10587.4\|MYH11_CHICK | UP |
| 136 | NP_001292602.1 | ribosome-recycling factor, mitochondrial [Gallus gallus] | 0.006148 | gi\|50757135\|ref\|XP_415395.1\| | UP |
| 137 | NP_001006302.1 | 45 kDa calcium-binding protein [Gallus gallus] | 0.03051 | gi\|55741731\|ref\|NP_001006302.1\| | UP |
| 138 | XP_015152979.1 | PREDICTED U4/U6.U5 small nuclear ribonucleoprotein 27 kDa protein [Gallus gallus] | 0.003856 | gi\|449269017\|gb\|EMC79827.1\| | UP |
| 139 | XP_415585.4 | PREDICTED dynein heavy chain 9, axonemal [Gallus gallus] | 0.001696 | gi\|363740657\|ref\|XP_415585.3\| | UP |
| 140 | XP_015144635.1 | PREDICTED collagen alpha-3(VI) chain isoform X7 [Gallus gallus] | 0.02809 | gi\|45382993\|ref\|NP_990865.1\| | UP |
| 141 | XP_015140314.1 | PREDICTED ras-related protein Rab-23 isoform X1 [Gallus gallus] | 0.01391 | gi\|158519801\|ref\|NP_001103530.1\| | UP |
| 142 | XP_015144781.1 | PREDICTED leucine-rich repeat flightless-interacting protein 1 isoform X34 [Gallus gallus] | 0.003149 | gi\|148708121\|gb\|EDL40068.1\| | UP |
| 143 | NP_001264684.1 | 60S ribosomal protein L31 [Gallus gallus] | 0.002861 | gi\|118084259\|ref\|XP_416909.2\| | UP |
| 144 | XP_015142275.1 | PREDICTED serine/threonine-protein phosphatase 6 regulatory subunit 3 isoform X6 [Gallus gallus] | 0.03671 | gi\|71894719\|ref\|NP_001026354.1\| | UP |
| 145 | XP_015133919.1 | PREDICTED UAP56-interacting factor isoform X2 [Gallus gallus] | 0.0006053 | gi\|363732750\|ref\|XP_420211.3\| | UP |
| 146 | XP_015153964.1 | PREDICTED atrial natriuretic peptide receptor 1-like, partial [Gallus gallus] | 0.0193 | gi\|344242035\|gb\|EGV98138.1\| | UP |
| 147 | XP_414478.4 | PREDICTED E3 ubiquitin/ISG15 ligase TRIM25 [Gallus gallus] | 0.01665 | gi\|363739076\|ref\|XP_414478.3\| | UP |
| 148 | XP_015135166.1 | PREDICTED myosin-5-like isoform X2 [Gallus gallus] | 0.006037 | gi\|465959370\|gb\|EMP28405.1\| | UP |
| 149 | XP_015151087.1 | PREDICTED lysosomal alpha-glucosidase [Gallus gallus] | 0.001555 | gi\|18693231\|dbj\|BAA25890.2\| | UP |
| 150 | NP_001025867.1 | nuclear distribution protein nudE-like 1 [Gallus gallus] | 0.008727 | gi\|71897361\|ref\|NP_001025867.1\| | UP |
| 151 | XP_015148816.1 | PREDICTED epidermal differentiation-specific protein-like [Gallus gallus] | 0.002842 | gi\|224066367\|ref\|XP_002187313.1\| | UP |
| 152 | XP_416537.3 | PREDICTED T-box transcription factor TBX15 [Gallus gallus] | 0.02491 | gi\|363728368\|ref\|XP_416537.3\| | UP |
| 153 | XP_015130509.1 | PREDICTED U1 small nuclear ribonucleoprotein 70 kDa-like, partial [Gallus gallus] | 0.0009798 | gi\|465998214\|gb\|EMP40554.1\| | UP |
| 154 | NP_989969.2 | follistatin-related protein 1 precursor [Gallus gallus] | 0.0437 | gi\|45382855\|ref\|NP_989969.1\| | UP |
| 155 | XP_003640794.1 | PREDICTED probable phospholipid-transporting ATPase IIB [Gallus gallus] | 0.03327 | gi\|363730313\|ref\|XP_003640794.1\| | UP |
| 156 | XP_004934590.1 | PREDICTED potassium voltage-gated channel subfamily E member 2 [Gallus gallus] | 0.02804 | gi\|118083866\|ref\|XP_425549.2\| | UP |
| 157 | BGI_novel_T007514 | gene=416214 [translate_table standard] | 0.03589 | - | UP |
| 158 | NP_001026067.1 | ras-related GTP-binding protein C [Gallus gallus] | 0.00802 | gi\|71894727\|ref\|NP_001026067.1\| | UP |
| 159 | NP_989967.1 | matrix-remodeling-associated protein 8 precursor [Gallus gallus] | 0.003905 | gi\|45382863\|ref\|NP_989967.1\| | UP |
| 160 | BGI_novel_T006144 | gene=396291 [translate_table standard] | 0.04133 | - | UP |
| 161 | XP_004943345.1 | PREDICTED BMP/retinoic acid-inducible neural-specific protein 3 [Gallus gallus] | 0.02797 | gi\|118094245\|ref\|XP_426633.2\| | DOWN |
| 162 | XP_015137325.1 | PREDICTED oxysterol-binding protein-related protein 3 isoform X6 [Gallus gallus] | 6.13E-05 | gi\|118085962\|ref\|XP_425992.2\| | DOWN |
| 163 | XP_425039.1 | PREDICTED fructose-1,6-bisphosphatase isozyme 2 [Gallus gallus] | 0.001709 | gi\|50762391\|ref\|XP_425039.1\| | DOWN |
| 164 | NP_990800.1 | pyruvate kinase PKM [Gallus gallus] | 0.03019 | gi\|45382651\|ref\|NP_990800.1\| | DOWN |
| 165 | XP_015151491.1 | PREDICTED DNA-directed RNA polymerase II subunit RPB11-a [Gallus gallus] | 0.01753 | gi\|5453932\|ref\|NP_006225.1\| | DOWN |
| 166 | XP_004944058.1 | PREDICTED gamma-tubulin complex component 4 isoform X4 [Gallus gallus] | 0.04171 | gi\|118096065\|ref\|XP_413958.2\| | DOWN |
| 167 | XP_015150680.1 | PREDICTED myosin-13 [Gallus gallus] | 0.0003696 | gi\|363740651\|ref\|XP_001231456.2\| | DOWN |
| 168 | XP_015152524.1 | PREDICTED polynucleotide 5'-hydroxyl-kinase NOL9 [Gallus gallus] | 0.02644 | gi\|363741919\|ref\|XP_001233522.2\| | DOWN |
| 169 | NP_001026534.1 | mediator of RNA polymerase II transcription subunit 24 [Gallus gallus] | 0.02292 | gi\|71897119\|ref\|NP_001026534.1\| | DOWN |
| 170 | BGI_novel_T005127 | gene=BGI_novel_G000072 [translate_table standard] | 0.00239 | gi\|363735918\|ref\|XP_421979.3\| | DOWN |
| 171 | XP_421619.3 | PREDICTED myozenin-1 isoform X1 [Gallus gallus] | 0.02078 | gi\|326923605\|ref\|XP_003208025.1\| | DOWN |
| 172 | XP_015136599.1 | PREDICTED obscurin [Gallus gallus] | 0.004951 | gi\|363729877\|ref\|XP_418501.3\| | DOWN |
| 173 | XP_418038.1 | PREDICTED C-_U-editing enzyme APOBEC-2 [Gallus gallus] | 0.0009381 | gi\|50760475\|ref\|XP_418038.1\| | DOWN |
| 174 | XP_413966.3 | PREDICTED chondroitin sulfate synthase 3 [Gallus gallus] | 0.003146 | gi\|363744614\|ref\|XP_413966.3\| | DOWN |
| 175 | NP_001026598.1 | guanine nucleotide-binding protein G(q) subunit alpha [Gallus gallus] | 0.00902 | gi\|71894823\|ref\|NP_001026598.1\| | DOWN |
| 176 | NP_001006395.1 | malate dehydrogenase, cytoplasmic isoform MDH1 [Gallus gallus] | 0.002796 | gi\|57530355\|ref\|NP_001006395.1\| | DOWN |
| 177 | XP_015131798.1 | PREDICTED PDZ and LIM domain protein 5 isoform X15 [Gallus gallus] | 0.01459 | gi\|71896681\|ref\|NP_001026320.1\| | DOWN |
| 178 | XP_015132769.1 | PREDICTED E3 SUMO-protein ligase PIAS2 isoform X4 [Gallus gallus] | 0.003752 | gi\|71894815\|ref\|NP_001025797.1\| | DOWN |
| 179 | NP_990850.1 | sarcoplasmic/endoplasmic reticulum calcium ATPase 1 [Gallus gallus] | 0.0142 | gi\|45382929\|ref\|NP_990850.1\| | DOWN |
| 180 | XP_015147554.1 | PREDICTED mothers against decapentaplegic homolog 3 isoform X1 [Gallus gallus] | 9.64E-06 | gi\|326926879\|ref\|XP_003209624.1\| | DOWN |
| 181 | XP_015152280.1 | PREDICTED myosin-7B [Gallus gallus] | 0.03456 | gi\|45383005\|ref\|NP_989918.1\| | DOWN |
| 182 | XP_418911.2 | PREDICTED magnesium transporter MRS2 homolog, mitochondrial [Gallus gallus] | 0.0001912 | gi\|118086357\|ref\|XP_418911.2\| | DOWN |
| 183 | XP_015149397.1 | PREDICTED gem-associated protein 5 [Gallus gallus] | 0.02132 | gi\|118097428\|ref\|XP_414574.2\| | DOWN |
| 184 | NP_990466.1 | M-protein, striated muscle [Gallus gallus] | 0.005327 | gi\|45384088\|ref\|NP_990466.1\| | DOWN |
| 185 | XP_015148734.1 | PREDICTED leiomodin-3 [Gallus gallus] | 0.00624 | gi\|326928126\|ref\|XP_003210234.1\| | DOWN |
| 186 | NP_001264396.1 | DNA-directed RNA polymerases I, II, and III subunit RPABC3 isoform 2 [Gallus gallus] | 0.02597 | gi\|403270066\|ref\|XP_003927019.1\| | DOWN |
| 187 | BGI_novel_T013034 | gene=107050762 [translate_table standard] | 5.25E-05 | gi\|345315624\|ref\|XP_001518327.2\| | DOWN |
| 188 | NP_001006160.2 | 7,8-dihydro-8-oxoguanine triphosphatase [Gallus gallus] | 0.01991 | gi\|164452937\|ref\|NP_001006160.2\| | DOWN |
| 189 | NP_001269225.1 | protein KTI12 homolog [Gallus gallus] | 0.003494 | gi\|118094549\|ref\|XP_422471.2\| | DOWN |
| 190 | XP_015130796.1 | PREDICTED protein-tyrosine sulfotransferase 2 isoform X1 [Gallus gallus] | 0.03244 | gi\|61098294\|ref\|NP_001012812.1\| | DOWN |
| 191 | XP_015154178.1 | PREDICTED voltage-dependent L-type calcium channel subunit alpha-1S isoform X1 [Gallus gallus] | 0.01961 | gi\|363742992\|ref\|XP_419259.3\| | DOWN |
| 192 | XP_015133019.1 | PREDICTED rapamycin-insensitive companion of mTOR isoform X2 [Gallus gallus] | 5.29E-05 | gi\|363744343\|ref\|XP_003643026.1\| | DOWN |
| 193 | XP_004939517.2 | PREDICTED probable leucine--tRNA ligase, mitochondrial isoform X1 [Gallus gallus] | 0.003435 | gi\|50732866\|ref\|XP_418801.1\| | DOWN |
| 194 | XP_015129142.1 | PREDICTED molybdate-anion transporter isoform X2 [Gallus gallus] | 0.003136 | gi\|363746034\|ref\|XP_003643503.1\| | DOWN |
| 195 | XP_015141354.1 | PREDICTED histone-lysine N-methyltransferase SMYD1 isoform X1 [Gallus gallus] | 0.003074 | gi\|326919603\|ref\|XP_003206069.1\| | DOWN |
| 196 | NP_001038124.1 | myosin-binding protein C, fast-type [Gallus gallus] | 0.02008 | gi\|113206110\|ref\|NP_001038124.1\| | DOWN |
| 197 | NP_001289036.1 | cytochrome c oxidase subunit NDUFA4 [Gallus gallus] | 0.00161 | gi\|363730012\|ref\|XP_001234601.2\| | DOWN |
| 198 | BGI_novel_T000374 | gene=418099 [translate_table standard] | 0.002487 | - | DOWN |
| 199 | NP_990191.2 | CD44 antigen precursor [Gallus gallus] | 0.04627 | gi\|5002373\|gb\|AAD37443.1\|AF153205_1 | DOWN |
| 200 | XP_015136481.1 | PREDICTED rho guanine nucleotide exchange factor 17 [Gallus gallus] | 0.0002491 | gi\|327281476\|ref\|XP_003225474.1\| | DOWN |
| 201 | NP_001264756.1 | myozenin-2 [Gallus gallus] | 0.01121 | gi\|118090320\|ref\|XP_420634.2\| | DOWN |
| 202 | BGI_novel_T005115 | gene=BGI_novel_G000060 [translate_table standard] | 0.00224 | gi\|363735918\|ref\|XP_421979.3\| | DOWN |
| 203 | XP_001233591.2 | PREDICTED uncharacterized protein LOC770260 isoform X2 [Gallus gallus] | 0.005945 | gi\|118098276\|ref\|XP_001233591.1\| | DOWN |
| 204 | XP_004947937.1 | PREDICTED cytosolic 5'-nucleotidase 1A isoform X4 [Gallus gallus] | 0.001094 | gi\|118101792\|ref\|XP_417822.2\| | DOWN |
| 205 | BGI_novel_T010193 | gene=419783 [translate_table standard] | 0.001071 | - | DOWN |
| 206 | XP_015136112.1 | PREDICTED COMM domain-containing protein 10 isoform X8 [Gallus gallus] | 0.002183 | gi\|224092134\|ref\|XP_002189795.1\| | DOWN |
| 207 | XP_015145352.1 | PREDICTED nebulin [Gallus gallus] | 0.01079 | gi\|363736047\|ref\|XP_422163.3\| | DOWN |
| 208 | XP_415846.2 | PREDICTED centromere protein V [Gallus gallus] | 0.0004645 | gi\|118100257\|ref\|XP_415846.2\| | DOWN |
| 209 | XP_422480.1 | PREDICTED enoyl-CoA hydratase domain-containing protein 2, mitochondrial [Gallus gallus] | 0.01661 | gi\|50751616\|ref\|XP_422480.1\| | DOWN |
| 210 | NP_001152787.1 | PREDICTED parvalbumin, muscle isoform X1 [Gallus gallus] | 0.01448 | gi\|226533801\|ref\|NP_001152787.1\| | DOWN |
| 211 | NP_990782.1 | triosephosphate isomerase [Gallus gallus] | 0.001717 | gi\|45382061\|ref\|NP_990782.1\| | DOWN |
| 212 | NP_001006296.1 | vesicle-associated membrane protein-associated protein B/C [Gallus gallus] | 0.03478 | gi\|57529406\|ref\|NP_001006296.1\| | DOWN |
| 213 | XP_421192.1 | PREDICTED vesicle transport through interaction with t-SNAREs homolog 1B [Gallus gallus] | 0.005386 | gi\|50748320\|ref\|XP_421192.1\| | DOWN |
| 214 | XP_015130086.1 | PREDICTED LOW QUALITY PROTEIN beta-enolase-like, partial [Gallus gallus] | 0.001187 | gi\|46048765\|ref\|NP_990450.1\| | DOWN |
| 215 | BGI_novel_T004701 | gene=423830 [translate_table standard] | 0.0004298 | gi\|118092890\|ref\|XP_001233069.1\| | DOWN |
| 216 | XP_015138832.1 | PREDICTED reticulon-4 isoform X5 [Gallus gallus] | 0.02453 | gi\|459642346\|ref\|NP_001264016.1\| | DOWN |
| 217 | BGI_novel_T001922 | gene=421083 [translate_table standard] | 0.01463 | gi\|344242514\|gb\|EGV98617.1\| | DOWN |
| 218 | XP_004935437.2 | PREDICTED disheveled-associated activator of morphogenesis 2 isoform X4 [Gallus gallus] | 0.00214 | gi\|363731815\|ref\|XP_419476.3\| | DOWN |
| 219 | XP_015129914.1 | PREDICTED glycogen phosphorylase, muscle form-like, partial [Gallus gallus] | 0.007979 | gi\|471402862\|ref\|XP_004383823.1\| | DOWN |
| 220 | NP_001273190.1 | LIM domain binding 3 [Gallus gallus] | 0.004016 | gi\|148692935\|gb\|EDL24882.1\| | DOWN |
| 221 | XP_416940.1 | PREDICTED protein ADP-ribosylarginine hydrolase-like protein 1 isoform X2 [Gallus gallus] | 0.004679 | gi\|50730512\|ref\|XP_416940.1\| | DOWN |
| 222 | XP_421891.4 | PREDICTED germinal-center associated nuclear protein [Gallus gallus] | 0.02512 | gi\|363735788\|ref\|XP_421891.3\| | DOWN |
| 223 | XP_420071.4 | PREDICTED 39S ribosomal protein L19, mitochondrial [Gallus gallus] | 0.01814 | gi\|363732486\|ref\|XP_420071.3\| | DOWN |
| 224 | NP_001116163.1 | thioredoxin reductase 2, mitochondrial [Gallus gallus] | 0.004932 | gi\|170015974\|ref\|NP_001116163.1\| | DOWN |
| 225 | BGI_novel_T004215 | gene=423258 [translate_table standard] | 0.01919 | - | DOWN |
| 226 | XP_015134942.1 | PREDICTED far upstream element-binding protein 3 isoform X4 [Gallus gallus] | 0.003777 | gi\|326930374\|ref\|XP_003211322.1\| | DOWN |
| 227 | XP_015154405.1 | PREDICTED neurofascin isoform X15 [Gallus gallus] | 0.02565 | gi\|38372283\|sp\|O42414.1\|NFASC_CHICK | DOWN |
| 228 | BGI_novel_T010899 | gene=419951 [translate_table standard] | 0.004739 | - | DOWN |
| 229 | XP_001232866.1 | PREDICTED histidine triad nucleotide-binding protein 3 [Gallus gallus] | 0.0001475 | gi\|118088593\|ref\|XP_001232866.1\| | DOWN |
| 230 | XP_015129776.1 | PREDICTED fructose-bisphosphate aldolase A-like, partial [Gallus gallus] | 0.001476 | gi\|149067832\|gb\|EDM17384.1\| | DOWN |
| 231 | XP_419073.2 | PREDICTED muscle-related coiled-coil protein [Gallus gallus] | 0.001112 | gi\|118086692\|ref\|XP_419073.2\| | DOWN |
| 232 | XP_015151589.1 | PREDICTED protein phosphatase Slingshot homolog 2 isoform X3 [Gallus gallus] | 0.02289 | gi\|363741338\|ref\|XP_415832.3\| | DOWN |
| 233 | NP_001027570.1 | eukaryotic translation elongation factor 1 alpha 2 [Gallus gallus] | 0.005542 | gi\|74048411\|ref\|NP_001027570.1\| | DOWN |
| 234 | BGI_novel_T000768 | gene=395664 [translate_table standard] | 0.0006575 | - | DOWN |
| 235 | NP_001006128.1 | glucose-6-phosphate isomerase [Gallus gallus] | 9.37E-06 | gi\|57524920\|ref\|NP_001006128.1\| | DOWN |
| 236 | XP_015149801.1 | PREDICTED transformation/transcription domain-associated protein isoform X4 [Gallus gallus] | 0.0001251 | gi\|449476158\|ref\|XP_002196759.2\| | DOWN |
| 237 | NP_990788.2 | proto-oncogene tyrosine-protein kinase Src [Gallus gallus] | 0.001087 | gi\|311771630\|ref\|NP_990788.2\| | DOWN |
| 238 | XP_015134075.1 | PREDICTED ATP-binding cassette sub-family B member 7, mitochondrial isoform X2 [Gallus gallus] | 0.007293 | gi\|50745926\|ref\|XP_420301.1\| | DOWN |
| 239 | XP_001234418.2 | PREDICTED proline-rich protein 33-like [Gallus gallus] | 0.002418 | gi\|118091311\|ref\|XP_001234418.1\| | DOWN |
| 240 | XP_015130558.1 | PREDICTED myosin-7 [Gallus gallus] | 0.0003547 | gi\|1814388\|gb\|AAB41889.1\| | DOWN |
| 241 | XP_015153400.1 | PREDICTED myomesin-3 [Gallus gallus] | 0.002413 | gi\|363742433\|ref\|XP_417838.3\| | DOWN |
| 242 | NP_001265026.1 | ATP synthase subunit gamma, mitochondrial isoform 2 [Gallus gallus] | 0.002158 | gi\|363727400\|ref\|XP_417296.2\| | DOWN |
| 243 | NP_001186105.1 | PREDICTED patched domain-containing protein 2 isoform X4 [Gallus gallus] | 9.35E-07 | gi\|312596920\|ref\|NP_001186105.1\| | DOWN |
| 244 | XP_015142062.1 | PREDICTED troponin T, fast skeletal muscle isoforms isoform X42 [Gallus gallus] | 0.001366 | gi\|326920134\|ref\|XP_003206330.1\| | DOWN |
| 245 | XP_015151416.1 | PREDICTED eosinophil peroxidase isoform X2 [Gallus gallus] | 0.03533 | gi\|118100016\|ref\|XP_415716.2\| | DOWN |
| 246 | XP_423728.2 | PREDICTED mast cell protease 1A [Gallus gallus] | 0.02925 | gi\|118103372\|ref\|XP_423728.2\| | DOWN |
| 247 | NP_989636.1 | glyceraldehyde-3-phosphate dehydrogenase [Gallus gallus] | 0.0008926 | gi\|46048961\|ref\|NP_989636.1\| | DOWN |
| 248 | NP_989857.1 | calsequestrin-2 precursor [Gallus gallus] | 0.01821 | gi\|46093996\|ref\|NP_989857.1\| | DOWN |
| 249 | XP_015130153.1 | PREDICTED ATP-dependent Clp protease proteolytic subunit, mitochondrial isoform X2 [Gallus gallus] | 0.006042 | gi\|345314392\|ref\|XP_001515048.2\| | DOWN |
| 250 | BGI_novel_T004732 | gene=423851 [translate_table standard] | 0.01023 | - | DOWN |
| 251 | XP_015146222.1 | PREDICTED polypyrimidine tract-binding protein 2 isoform X7 [Gallus gallus] | 0.004439 | gi\|440902651\|gb\|ELR53420.1\| | DOWN |
| 252 | XP_015142808.1 | PREDICTED exonuclease 3'-5' domain-containing protein 2 isoform X2 [Gallus gallus] | 0.001552 | gi\|314122235\|ref\|NP_001186629.1\| | DOWN |
| 253 | BGI_novel_T008705 | gene=417352 [translate_table standard] | 0.00175 | - | DOWN |
| 254 | BGI_novel_T005109 | gene=BGI_novel_G000054 [translate_table standard] | 0.002073 | gi\|363735918\|ref\|XP_421979.3\| | DOWN |
| 255 | NP_001161224.1 | myoglobin [Gallus gallus] | 0.003692 | gi\|268607704\|ref\|NP_001161224.1\| | DOWN |
| 256 | XP_015136977.1 | PREDICTED protein MON2 homolog isoform X3 [Gallus gallus] | 0.03296 | gi\|313760707\|ref\|NP_001186534.1\| | DOWN |
| 257 | XP_421618.3 | PREDICTED synaptopodin 2-like protein isoform X1 [Gallus gallus] | 0.02323 | gi\|363735650\|ref\|XP_421618.3\| | DOWN |
| 258 | BGI_novel_T005336 | gene=424232 [translate_table standard] | 0.01988 | - | DOWN |
| 259 | NP_989559.2 | myosin-3 [Gallus gallus] | 0.04971 | gi\|45383668\|ref\|NP_989559.1\| | DOWN |
| 260 | BGI_novel_T002707 | gene=101750381 [translate_table standard] | 0.0003321 | - | DOWN |
| 261 | BGI_novel_T003916 | gene=423043 [translate_table standard] | 0.001307 | - | DOWN |
| 262 | NP_001075172.1 | histone H1.11R [Gallus gallus] | 0.005206 | gi\|126165252\|ref\|NP_001075172.1\| | DOWN |
| 263 | NP_990440.1 | adenylate kinase isoenzyme 1 [Gallus gallus] | 0.007588 | gi\|46048771\|ref\|NP_990440.1\| | DOWN |
| 264 | NP_990652.1 | aspartate aminotransferase, cytoplasmic [Gallus gallus] | 0.03353 | gi\|45384348\|ref\|NP_990652.1\| | DOWN |
| 265 | XP_015130270.1 | PREDICTED C-type lectin domain family 4 member G-like, partial [Gallus gallus] | 0.02435 | gi\|466001447\|gb\|EMP41501.1\| | DOWN |
| 266 | XP_015134524.1 | PREDICTED mannose-6-phosphate isomerase isoform X1 [Gallus gallus] | 0.006833 | gi\|363737541\|ref\|XP_001233064.2\| | DOWN |
| 267 | BGI_novel_T005119 | gene=BGI_novel_G000064 [translate_table standard] | 0.00309 | gi\|363735918\|ref\|XP_421979.3\| | DOWN |
| 268 | XP_414589.2 | PREDICTED myozenin-3 [Gallus gallus] | 0.01725 | gi\|118097461\|ref\|XP_414589.2\| | DOWN |
| 269 | NP_990654.1 | alpha-actinin-2 [Gallus gallus] | 0.003843 | gi\|46048687\|ref\|NP_990654.1\| | DOWN |
| 270 | XP_004946569.1 | PREDICTED protein unc-45 homolog B [Gallus gallus] | 0.009075 | gi\|363741087\|ref\|XP_415774.3\| | DOWN |
| 271 | XP_004935188.2 | PREDICTED 3-oxo-5-alpha-steroid 4-dehydrogenase 1 [Gallus gallus] | 0.004828 | gi\|363730576\|ref\|XP_001233788.2\| | DOWN |
| 272 | XP_015154260.1 | PREDICTED synaptophysin-like protein 2 isoform X2 [Gallus gallus] | 0.007449 | gi\|363745794\|ref\|XP_003643420.1\| | DOWN |
| 273 | XP_015147474.1 | PREDICTED pleckstrin homology domain-containing family A member 5 isoform X19 [Gallus gallus] | 4.43E-05 | gi\|363728160\|ref\|XP_416414.3\| | DOWN |
| 274 | NP_001107181.1 | myosin, heavy chain 1C, skeletal muscle [Gallus gallus] | 0.0002165 | gi\|165973976\|ref\|NP_001107181.1\| | DOWN |
| 275 | NP_001026727.1 | phosphoglycerate mutase 1 [Gallus gallus] | 0.0004723 | gi\|71895985\|ref\|NP_001026727.1\| | DOWN |
| 276 | XP_015153073.1 | PREDICTED lysine-specific histone demethylase 1A isoform X2 [Gallus gallus] | 5.36E-06 | gi\|3043726\|dbj\|BAA25527.1\| | DOWN |
| 277 | XP_015139068.1 | PREDICTED 1-phosphatidylinositol 4,5-bisphosphate phosphodiesterase beta-1 [Gallus gallus] | 0.002511 | gi\|363731813\|ref\|XP_425261.3\| | DOWN |
| 278 | NP_001258902.1 | sarcoplasmic/endoplasmic reticulum calcium ATPase 2 isoform 1 [Gallus gallus] | 0.01161 | gi\|430736679\|ref\|NP_001258902.1\| | DOWN |
| 279 | XP_004940659.1 | PREDICTED phosphorylase b kinase regulatory subunit alpha, skeletal muscle isoform isoform X5 [Gallus gallus] | 0.001282 | gi\|363732701\|ref\|XP_003641138.1\| | DOWN |
| 280 | XP_015142788.1 | PREDICTED ryanodine receptor 3 isoform X11 [Gallus gallus] | 0.0421 | gi\|46048744\|ref\|NP_996757.1\| | DOWN |
| 281 | XP_015139201.1 | PREDICTED exportin-5 isoform X3 [Gallus gallus] | 0.0004319 | gi\|118088066\|ref\|XP_419501.2\| | DOWN |
| 282 | NP_001264844.1 | ELMO domain-containing protein 2 [Gallus gallus] | 0.0002606 | gi\|118089918\|ref\|XP_420415.2\| | DOWN |
| 283 | NP_001264343.1 | 39S ribosomal protein L44, mitochondrial precursor [Gallus gallus] | 0.03922 | gi\|50752008\|ref\|XP_422612.1\| | DOWN |
| 284 | XP_004939354.1 | PREDICTED sterile alpha motif domain-containing protein 9-like [Gallus gallus] | 0.004021 | gi\|118085828\|ref\|XP_418660.2\| | DOWN |
| 285 | NP_001183965.1 | transcriptional adapter 1 [Gallus gallus] | 0.003892 | gi\|334358894\|ref\|NP_001183965.1\| | DOWN |
| 286 | NP_990588.2 | proto-oncogene tyrosine-protein kinase ROS precursor [Gallus gallus] | 0.0004072 | gi\|160333842\|ref\|NP_990588.2\| | DOWN |
| 287 | XP_015151569.1 | PREDICTED TBC1 domain family member 23 isoform X1 [Gallus gallus] | 0.001699 | gi\|326912978\|ref\|XP_003202820.1\| | DOWN |
| 288 | NP_001265029.1 | adipogenesis associated, Mth938 domain containing [Gallus gallus] | 0.004033 | gi\|50808963\|ref\|XP_424622.1\| | DOWN |
| 289 | XP_015128481.1 | PREDICTED EH domain-binding protein 1-like protein 1 isoform X4 [Gallus gallus] | 0.005935 | gi\|62420307\|gb\|AAX82024.1\| | DOWN |
| 290 | BGI_novel_T005132 | gene=BGI_novel_G000077 [translate_table standard] | 0.001672 | gi\|363735918\|ref\|XP_421979.3\| | DOWN |
| 291 | NP_989747.1 | neuronal acetylcholine receptor subunit alpha-3 precursor [Gallus gallus] | 0.0007554 | gi\|56606123\|ref\|NP_989747.1\| | DOWN |
| 292 | NP_989625.1 | calcium/calmodulin-dependent protein kinase type II subunit beta [Gallus gallus] | 0.000671 | gi\|46048967\|ref\|NP_989625.1\| | DOWN |
| 293 | XP_004934896.1 | PREDICTED troponin I, slow skeletal muscle [Gallus gallus] | 0.04798 | gi\|363743002\|ref\|XP_419242.3\| | DOWN |
| 294 | XP_416732.1 | PREDICTED SH3 domain-binding glutamic acid-rich protein [Gallus gallus] | 0.03275 | gi\|50729975\|ref\|XP_416732.1\| | DOWN |
| 295 | XP_015130132.1 | PREDICTED glycogen phosphorylase, muscle form-like [Gallus gallus] | 0.01753 | gi\|327290807\|ref\|XP_003230113.1\| | DOWN |
| 296 | BGI_novel_T005123 | gene=BGI_novel_G000068 [translate_table standard] | 0.002113 | gi\|363735918\|ref\|XP_421979.3\| | DOWN |
| 297 | BGI_novel_T005107 | gene=BGI_novel_G000052 [translate_table standard] | 0.004868 | gi\|363735918\|ref\|XP_421979.3\| | DOWN |
| 298 | XP_004942386.1 | PREDICTED WD repeat-containing protein 11 isoform X4 [Gallus gallus] | 0.007336 | gi\|118093113\|ref\|XP_421795.2\| | DOWN |
| 299 | XP_422010.1 | PREDICTED kelch-like protein 41 [Gallus gallus] | 0.01405 | gi\|50750473\|ref\|XP_422010.1\| | DOWN |
| 300 | XP_015141125.1 | PREDICTED alcohol dehydrogenase 1-like isoform X2 [Gallus gallus] | 0.0004371 | gi\|45384164\|ref\|NP_990423.1\| | DOWN |
| 301 | NP_001035732.1 | histone H1.01 [Gallus gallus] | 0.0359 | gi\|449279166\|gb\|EMC86812.1\| | DOWN |
| 302 | NP_989554.3 | PREDICTED ATP-dependent 6-phosphofructokinase, muscle type isoform X1 [Gallus gallus] | 0.01161 | gi\|347800728\|ref\|NP_989554.2\| | DOWN |
| 303 | XP_415252.3 | PREDICTED melanotransferrin-like isoform X2 [Gallus gallus] | 0.0003672 | gi\|363740145\|ref\|XP_415252.3\| | DOWN |
| 304 | NP_001289056.1 | apolipoprotein C-III precursor [Gallus gallus] | 0.007628 | gi\|363742557\|ref\|XP_003642651.1\| | DOWN |
| 305 | XP_015153678.1 | PREDICTED tripartite motif-containing protein 29 isoform X6 [Gallus gallus] | 1.11E-05 | gi\|363742535\|ref\|XP_417892.3\| | DOWN |
| 306 | BGI_novel_T001718 | gene=428476 [translate_table standard] | 0.006815 | gi\|363730349\|ref\|XP_426033.3\| | DOWN |
| 307 | XP_001234380.3 | PREDICTED ADP-ribosylation factor-like protein 9 [Gallus gallus] | 0.01317 | gi\|363733416\|ref\|XP_001234380.2\| | DOWN |
| 308 | BGI_novel_T004860 | gene=423960 [translate_table standard] | 0.01403 | - | DOWN |
| 309 | XP_015143471.1 | PREDICTED LIM domain-binding protein 3 isoform X5 [Gallus gallus] | 0.005676 | gi\|327277117\|ref\|XP_003223312.1\| | DOWN |
| 310 | XP_015139145.1 | PREDICTED N-lysine methyltransferase SMYD2 isoform X1 [Gallus gallus] | 0.027 | gi\|50740296\|ref\|XP_419420.1\| | DOWN |
| 311 | XP_421597.1 | PREDICTED apoptosis-inducing factor 2 [Gallus gallus] | 4.37E-06 | gi\|50749348\|ref\|XP_421597.1\| | DOWN |
| 312 | NP_001264383.1 | ral GTPase-activating protein subunit alpha-1 isoform 5 [Gallus gallus] | 0.001862 | gi\|363734121\|ref\|XP_003641344.1\| | DOWN |
| 313 | XP_004943549.1 | PREDICTED polyhomeotic-like protein 3 isoform X4 [Gallus gallus] | 0.0123 | gi\|363737247\|ref\|XP_426714.3\| | DOWN |
| 314 | XP_015153259.1 | PREDICTED four and a half LIM domains protein 3 isoform X2 [Gallus gallus] | 0.03878 | gi\|50759730\|ref\|XP_417754.1\| | DOWN |
| 315 | XP_004939317.1 | PREDICTED phosphotriesterase-related protein [Gallus gallus] | 1.77E-06 | gi\|50732421\|ref\|XP_418628.1\| | DOWN |
| 316 | XP_015134723.1 | PREDICTED prolyl 3-hydroxylase OGFOD1 isoform X1 [Gallus gallus] | 3.06E-06 | gi\|363738080\|ref\|XP_414062.3\| | DOWN |
| 317 | NP_001033782.2 | phosphoglucomutase-1 [Gallus gallus] | 0.006086 | gi\|84619526\|ref\|NP_001033782.1\| | DOWN |
| 318 | XP_004943912.1 | PREDICTED cytosolic carboxypeptidase 4 isoform X2 [Gallus gallus] | 0.0077 | gi\|363737598\|ref\|XP_425080.3\| | DOWN |
| 319 | XP_015151151.1 | PREDICTED myosin-7 isoform X2 [Gallus gallus] | 0.003508 | gi\|50838836\|ref\|NP_001001302.1\| | DOWN |
| 320 | XP_015128486.1 | PREDICTED uncharacterized protein LOC101751955 [Gallus gallus] | 0.0002781 | gi\|291384923\|ref\|XP_002709124.1\| | DOWN |
| 321 | NP_990748.1 | troponin I, fast skeletal muscle [Gallus gallus] | 0.01857 | gi\|45382253\|ref\|NP_990748.1\| | DOWN |
| 322 | BGI_novel_T001981 | gene=421142 [translate_table standard] | 0.0001677 | gi\|326917678\|ref\|XP_003205123.1\| | DOWN |
| 323 | BGI_novel_T005131 | gene=BGI_novel_G000076 [translate_table standard] | 0.0008557 | gi\|363735918\|ref\|XP_421979.3\| | DOWN |
| 324 | NP_990020.1 | histidine triad nucleotide-binding protein 1 [Gallus gallus] | 0.001197 | gi\|45382717\|ref\|NP_990020.1\| | DOWN |
| 325 | NP_001264340.1 | carbonic anhydrase III, muscle specific [Gallus gallus] | 1.33E-05 | gi\|50731674\|ref\|XP_418319.1\| | DOWN |
| 326 | NP_001007964.1 | importin subunit alpha-3 [Gallus gallus] | 0.003148 | gi\|56118970\|ref\|NP_001007964.1\| | DOWN |
| 327 | BGI_novel_T005911 | gene=424714 [translate_table standard] | 0.002934 | gi\|465970425\|gb\|EMP32391.1\| | DOWN |
| 328 | BGI_novel_T002069 | gene=420277 [translate_table standard] | 0.01955 | - | DOWN |
| 329 | BGI_novel_T011403 | gene=100858799 [translate_table standard] | 0.01105 | gi\|194384492\|dbj\|BAG59406.1\| | DOWN |
| 330 | XP_004941703.1 | PREDICTED cytosolic phospholipase A2 epsilon isoform X2 [Gallus gallus] | 0.00119 | gi\|363734442\|ref\|XP_001234838.2\| | DOWN |
| 331 | XP_004935061.1 | PREDICTED AMP deaminase 1 isoform X3 [Gallus gallus] | 1.34E-05 | gi\|363743097\|ref\|XP_003642776.1\| | DOWN |
| 332 | NP_990017.1 | steroidogenic acute regulatory protein, mitochondrial precursor [Gallus gallus] | 0.008597 | gi\|45382719\|ref\|NP_990017.1\| | DOWN |
| 333 | XP_015153271.1 | PREDICTED nucleolar GTP-binding protein 2 [Gallus gallus] | 0.0009158 | gi\|363742270\|ref\|XP_417761.2\| | DOWN |
| 334 | BGI_novel_T007225 | gene=769533 [translate_table standard] | 0.002532 | - | DOWN |
| 335 | XP_004944071.1 | PREDICTED protein NDRG4 isoform X5 [Gallus gallus] | 0.001206 | gi\|363737958\|ref\|XP_003641929.1\| | DOWN |
| 336 | XP_015155031.1 | PREDICTED telethonin [Gallus gallus] | 0.01326 | gi\|327275457\|ref\|XP_003222490.1\| | DOWN |
| 337 | XP_015129507.1 | PREDICTED ryanodine receptor 1-like, partial [Gallus gallus] | 0.007141 | gi\|163961395\|gb\|ABY50125.1\| | DOWN |
| 338 | XP_015142342.1 | PREDICTED smoothelin-like protein 1 isoform X2 [Gallus gallus] | 0.0228 | gi\|327260382\|ref\|XP_003215013.1\| | DOWN |
| 339 | NP_001277483.1 | band 3 anion transport protein [Gallus gallus] | 0.000413 | gi\|114786\|sp\|P15575.1\|B3AT_CHICK | DOWN |
| 340 | NP_990507.1 | alpha-crystallin B chain [Gallus gallus] | 0.006156 | gi\|45384008\|ref\|NP_990507.1\| | DOWN |
| 341 | BGI_novel_T005116 | gene=BGI_novel_G000061 [translate_table standard] | 0.006733 | gi\|148921373\|dbj\|BAF64430.1\| | DOWN |
| 342 | XP_015157102.1 | PREDICTED LOW QUALITY PROTEIN nebulin-like, partial [Gallus gallus] | 0.01305 | gi\|363736047\|ref\|XP_422163.3\| | DOWN |
| 343 | XP_003642358.1 | PREDICTED myosin heavy chain, skeletal muscle, adult isoform X2 [Gallus gallus] | 0.03084 | gi\|363740639\|ref\|XP_003642358.1\| | DOWN |
| 344 | XP_420194.1 | PREDICTED FUN14 domain-containing protein 2 [Gallus gallus] | 0.006681 | gi\|50745672\|ref\|XP_420194.1\| | DOWN |
| 345 | BGI_novel_T012805 | gene=429272 [translate_table standard] | 0.002239 | gi\|449265659\|gb\|EMC76820.1\| | DOWN |
| 346 | XP_001231979.2 | PREDICTED RWD domain-containing protein 4 isoform X2 [Gallus gallus] | 0.001748 | gi\|118090093\|ref\|XP_001231979.1\| | DOWN |
| 347 | XP_015142887.1 | PREDICTED DENN domain-containing protein 2A isoform X2 [Gallus gallus] | 0.000259 | gi\|224095593\|ref\|XP_002197636.1\| | DOWN |
| 348 | XP_003642396.1 | PREDICTED dual specificity mitogen-activated protein kinase kinase 6 isoform X2 [Gallus gallus] | 0.0003906 | gi\|363740834\|ref\|XP_003642396.1\| | DOWN |
| 349 | XP_004936247.1 | PREDICTED actin-binding LIM protein 2 isoform X13 [Gallus gallus] | 0.0001994 | gi\|363733606\|ref\|XP_420811.3\| | DOWN |
| 350 | NP_001107598.1 | cytochrome c oxidase assembly protein COX15 homolog [Gallus gallus] | 0.004052 | gi\|166295222\|ref\|NP_001107598.1\| | DOWN |
| 351 | XP_015148167.1 | PREDICTED alpha-2-macroglobulin [Gallus gallus] | 0.02206 | gi\|363728304\|ref\|XP_416476.3\| | DOWN |
| 352 | XP_015132976.1 | PREDICTED histidine triad nucleotide-binding protein 2, mitochondrial [Gallus gallus] | 0.0002658 | gi\|363744316\|ref\|XP_003643021.1\| | DOWN |
| 353 | XP_015132718.1 | PREDICTED talin-1 isoform X2 [Gallus gallus] | 0.0004504 | gi\|45383127\|ref\|NP_989854.1\| | DOWN |
| 354 | XP_004940735.2 | PREDICTED four and a half LIM domains protein 1 isoform X1 [Gallus gallus] | 0.03593 | gi\|363732773\|ref\|XP_001234114.2\| | DOWN |
| 355 | NP_001004375.1 | hemoglobin subunit alpha-D [Gallus gallus] | 0.01746 | gi\|52138645\|ref\|NP_001004375.1\| | DOWN |
| 356 | XP_422262.2 | PREDICTED torsin-1A-interacting protein 2 isoform X2 [Gallus gallus] | 0.00181 | gi\|118094140\|ref\|XP_422262.2\| | DOWN |
| 357 | XP_015140913.1 | PREDICTED PDZ and LIM domain protein 3 isoform X1 [Gallus gallus] | 0.01054 | gi\|449269876\|gb\|EMC80616.1\| | DOWN |
| 358 | NP_990838.1 | creatine kinase M-type [Gallus gallus] | 0.001774 | gi\|45382875\|ref\|NP_990838.1\| | DOWN |
| 359 | XP_004934477.2 | PREDICTED P2X purinoceptor 2 [Gallus gallus] | 0.02215 | gi\|327284265\|ref\|XP_003226859.1\| | DOWN |
| 360 | NP_001034694.1 | 5'-AMP-activated protein kinase catalytic subunit alpha-2 [Gallus gallus] | 0.0001908 | gi\|88853851\|ref\|NP_001034694.1\| | DOWN |
| 361 | XP_003641142.1 | PREDICTED 60S ribosomal protein L36a [Gallus gallus] | 0.003855 | gi\|363732720\|ref\|XP_003641142.1\| | DOWN |
| 362 | NP_001026292.1 | PREDICTED vesicle-associated membrane protein 7 isoform X1 [Gallus gallus] | 0.007284 | gi\|71895505\|ref\|NP_001026292.1\| | DOWN |
| 363 | NP_001026234.1 | actin, alpha skeletal muscle [Gallus gallus] | 0.003342 | gi\|4501881\|ref\|NP_001091.1\| | DOWN |
| 364 | NP_001026015.1 | protein AAR2 homolog [Gallus gallus] | 0.005223 | gi\|71895049\|ref\|NP_001026015.1\| | DOWN |
| 365 | XP_015143164.1 | PREDICTED myosin-binding protein C, slow-type isoform X4 [Gallus gallus] | 0.002249 | gi\|363728127\|ref\|XP_416332.3\| | DOWN |
| 366 | BGI_novel_T005393 | gene=424281 [translate_table standard] | 0.01079 | gi\|326923067\|ref\|XP_003207763.1\| | DOWN |
| 367 | XP_015149207.1 | PREDICTED calcium/calmodulin-dependent protein kinase type II subunit alpha isoform X3 [Gallus gallus] | 0.001202 | gi\|224067712\|ref\|XP_002198859.1\| | DOWN |
| 368 | XP_015136728.1 | PREDICTED GTP-binding protein Rheb isoform X9 [Gallus gallus] | 0.03312 | gi\|148671142\|gb\|EDL03089.1\| | DOWN |
| 369 | NP_001264339.1 | carbonic anhydrase III-like [Gallus gallus] | 0.04037 | gi\|50731676\|ref\|XP_418320.1\| | DOWN |
| 370 | XP_015135613.1 | PREDICTED multiple PDZ domain protein isoform X17 [Gallus gallus] | 0.01937 | gi\|343469213\|gb\|AEM43815.1\| | DOWN |
| 371 | XP_015133420.1 | PREDICTED myomesin-1 isoform X1 [Gallus gallus] | 0.005645 | gi\|45384434\|ref\|NP_990290.1\| | DOWN |
| 372 | XP_015129515.1 | PREDICTED LOW QUALITY PROTEIN glucosylceramidase-like, partial [Gallus gallus] | 0.03118 | gi\|449510911\|ref\|XP_004175596.1\| | DOWN |
| 373 | XP_004949628.1 | PREDICTED tumor suppressor candidate gene 1 protein [Gallus gallus] | 0.00197 | gi\|114623958\|ref\|XP_001153427.1\| | DOWN |
| 374 | XP_015130551.1 | PREDICTED glycogen [starch] synthase, muscle, partial [Gallus gallus] | 0.02074 | gi\|28569852\|dbj\|BAC57898.1\| | DOWN |
| 375 | BGI_novel_T013254 | gene=BGI_novel_G000571 [translate_table standard] | 0.01182 | gi\|149053074\|gb\|EDM04891.1\| | DOWN |
| 376 | BGI_novel_T007857 | gene=396269 [translate_table standard] | 0.008648 | gi\|326929327\|ref\|XP_003210818.1\| | DOWN |
| 377 | XP_424407.2 | PREDICTED DNA polymerase beta isoform X2 [Gallus gallus] | 0.003361 | gi\|118101412\|ref\|XP_424407.2\| | DOWN |
| 378 | XP_425758.4 | PREDICTED LOW QUALITY PROTEIN anthrax toxin receptor 1 [Gallus gallus] | 0.0004191 | gi\|363742080\|ref\|XP_425758.3\| | DOWN |
| 379 | NP_001006354.1 | nucleolar GTP-binding protein 1 [Gallus gallus] | 3.41E-05 | gi\|57530676\|ref\|NP_001006354.1\| | DOWN |
| 380 | XP_015135727.1 | PREDICTED intracellular hyaluronan-binding protein 4 isoform X1 [Gallus gallus] | 0.0003245 | gi\|45382669\|ref\|NP_990036.1\| | DOWN |
| 381 | XP_422450.1 | PREDICTED fatty-acid amide hydrolase 1 [Gallus gallus] | 0.002079 | gi\|50751550\|ref\|XP_422450.1\| | DOWN |
| 382 | XP_015157143.1 | PREDICTED titin-like [Gallus gallus] | 0.006958 | gi\|363735918\|ref\|XP_421979.3\| | DOWN |
