# Supplementary material for: Combined transcriptomics and proteomics forecast analysis for potential genes regulating the Columbian plumage color in chickens
Source: PLoS One. 2019 Nov 6;14(11):e0210850. doi: 10.1371/journal.pone.0210850 (PMC6834273; doi:10.1371/journal.pone.0210850)
Supplement: S5 Table — (DOCX) [file pone.0210850.s006.docx]

**Supplementary Table 5. Correlation DEPS and DEGs.**

| **NO** | **Protein ID** | **Protein Description** | **Protein Sig** | **Gene ID** | **Gene Sig** | **UP/**  **DOWN** |
| --- | --- | --- | --- | --- | --- | --- |
| 1 | XP_421619.3 | PREDICTED myozenin-1 isoform X1 [Gallus gallus] | * | 423744 | 0.918837519 | DOWN |
| 2 | XP_418038.1 | PREDICTED C-_U-editing enzyme APOBEC-2 [Gallus gallus] | * | 419915 | 0.892920285 | DOWN |
| 3 | NP_990850.1 | sarcoplasmic/endoplasmic reticulum calcium ATPase 1 [Gallus gallus] | * | 396528 | 0.926543065 | DOWN |
| 4 | XP_015152280.1 | PREDICTED myosin-7B [Gallus gallus] | * | 395279 | 0.887788469 | DOWN |
| 5 | NP_990466.1 | M-protein, striated muscle [Gallus gallus] | * | 396034 | 0.802964875 | DOWN |
| 6 | XP_015154178.1 | PREDICTED voltage-dependent L-type calcium channel subunit alpha-1S isoform X1 [Gallus gallus] | * | 395985 | 0.827317531 | DOWN |
| 7 | XP_015141354.1 | PREDICTED histone-lysine N-methyltransferase SMYD1 isoform X1 [Gallus gallus] | * | 373960 | 0.904920598 | DOWN |
| 8 | NP_001038124.1 | myosin-binding protein C, fast-type [Gallus gallus] | * | 425457 | 0.90140812 | DOWN |
| 9 | BGI_novel_T000374 | gene=418099 [translate_table standard] | * | 418099 | 0.858718611 | DOWN |
| 10 | NP_001264756.1 | myozenin-2 [Gallus gallus] | * | 422682 | 0.889446922 | DOWN |
| 11 | XP_001233591.2 | PREDICTED uncharacterized protein LOC770260 isoform X2 [Gallus gallus] | * | 770260 | 0.899514981 | DOWN |
| 12 | XP_015145352.1 | PREDICTED nebulin [Gallus gallus] | * | 374027 | 0.85914887 | DOWN |
| 13 | NP_001152787.1 | PREDICTED parvalbumin, muscle isoform X1 [Gallus gallus] | * | 396459 | 0.841711648 | DOWN |
| 14 | XP_015130086.1 | PREDICTED LOW QUALITY PROTEIN beta-enolase-like, partial [Gallus gallus] | * | 107050916 | 0.867057811 | DOWN |
| 15 | XP_015129914.1 | PREDICTED glycogen phosphorylase, muscle form-like, partial [Gallus gallus] | * | 107050715 | 0.924673394 | DOWN |
| 16 | NP_001273190.1 | LIM domain binding 3 [Gallus gallus] | * | 423610 | 0.880106391 | DOWN |
| 17 | XP_416940.1 | PREDICTED protein ADP-ribosylarginine hydrolase-like protein 1 isoform X2 [Gallus gallus] | * | 418741 | 0.895470547 | DOWN |
| 18 | XP_015129776.1 | PREDICTED fructose-bisphosphate aldolase A-like, partial [Gallus gallus] | * | 107050559 | 0.886114371 | DOWN |
| 19 | NP_001027570.1 | eukaryotic translation elongation factor 1 alpha 2 [Gallus gallus] | * | 419244 | 0.912610498 | DOWN |
| 20 | XP_001234418.2 | PREDICTED proline-rich protein 33-like [Gallus gallus] | * | 771119 | 0.909121489 | DOWN |
| 21 | XP_015130558.1 | PREDICTED myosin-7 [Gallus gallus] | * | 396063 | 0.87842447 | DOWN |
| 22 | XP_015142062.1 | PREDICTED troponin T, fast skeletal muscle isoforms isoform X42 [Gallus gallus] | * | 395761 | 0.920308222 | DOWN |
| 23 | NP_989857.1 | calsequestrin-2 precursor [Gallus gallus] | * | 395198 | 0.879989048 | DOWN |
| 24 | NP_001161224.1 | myoglobin [Gallus gallus] | * | 418056 | 0.926151921 | DOWN |
| 25 | XP_421618.3 | PREDICTED synaptopodin 2-like protein isoform X1 [Gallus gallus] | * | 423743 | 0.815137292 | DOWN |
| 26 | NP_989559.2 | myosin-3 [Gallus gallus] | * | 374069 | 0.815966518 | DOWN |
| 27 | NP_990440.1 | adenylate kinase isoenzyme 1 [Gallus gallus] | * | 396002 | 0.831831338 | DOWN |
| 28 | XP_015154260.1 | PREDICTED synaptophysin-like protein 2 isoform X2 [Gallus gallus] | * | 100858920 | 0.847688336 | DOWN |
| 29 | NP_001107181.1 | myosin, heavy chain 1C, skeletal muscle [Gallus gallus] | * | 417310 | 0.887193929 | DOWN |
| 30 | XP_004934896.1 | PREDICTED troponin I, slow skeletal muscle [Gallus gallus] | * | 421161 | 0.915301572 | DOWN |
| 31 | XP_015130132.1 | PREDICTED glycogen phosphorylase, muscle form-like [Gallus gallus] | * | 107050974 | 0.904193069 | DOWN |
| 32 | XP_422010.1 | PREDICTED kelch-like protein 41 [Gallus gallus] | * | 424164 | 0.830548385 | DOWN |
| 33 | NP_989554.3 | PREDICTED ATP-dependent 6-phosphofructokinase, muscle type isoform X1 [Gallus gallus] | * | 374064 | 0.909481342 | DOWN |
| 34 | XP_015143471.1 | PREDICTED LIM domain-binding protein 3 isoform X5 [Gallus gallus] | * | 423610 | 0.880106391 | DOWN |
| 35 | XP_015153259.1 | PREDICTED four and a half LIM domains protein 3 isoform X2 [Gallus gallus] | * | 419607 | 0.813517954 | DOWN |
| 36 | NP_001033782.2 | phosphoglucomutase-1 [Gallus gallus] | * | 424691 | 0.80246421 | DOWN |
| 37 | NP_990748.1 | troponin I, fast skeletal muscle [Gallus gallus] | * | 396386 | 0.928459673 | DOWN |
| 38 | NP_001264340.1 | carbonic anhydrase III, muscle specific [Gallus gallus] | * | 420208 | 0.85860909 | DOWN |
| 39 | XP_015155031.1 | PREDICTED telethonin [Gallus gallus] | * | 107055299 | 0.924000626 | DOWN |
| 40 | XP_015142342.1 | PREDICTED smoothelin-like protein 1 isoform X2 [Gallus gallus] | * | 101750657 | 0.868817961 | DOWN |
| 41 | NP_990507.1 | alpha-crystallin B chain [Gallus gallus] | * | 396089 | 0.871462098 | DOWN |
| 42 | XP_003642358.1 | PREDICTED myosin heavy chain, skeletal muscle, adult isoform X2 [Gallus gallus] | * | 427789 | 0.888633341 | DOWN |
| 43 | BGI_novel_T012805 | gene=429272 [translate_table standard] | * | 429272 | 0.851372917 | DOWN |
| 44 | XP_004940735.2 | PREDICTED four and a half LIM domains protein 1 isoform X1 [Gallus gallus] | * | 770787 | 0.872173981 | DOWN |
| 45 | NP_990838.1 | creatine kinase M-type [Gallus gallus] | * | 396507 | 0.921286083 | DOWN |
| 46 | NP_001026234.1 | actin, alpha skeletal muscle [Gallus gallus] | * | 421534 | 0.908683408 | DOWN |
| 47 | XP_015143164.1 | PREDICTED myosin-binding protein C, slow-type isoform X4 [Gallus gallus] | * | 418099 | 0.858718611 | DOWN |
| 48 | XP_015133420.1 | PREDICTED myomesin-1 isoform X1 [Gallus gallus] | * | 395805 | 0.801916608 | DOWN |
| 49 | BGI_novel_T007857 | gene=396269 [translate_table standard] | * | 396269 | 0.826175389 | DOWN |
